# Favorable safety and immunogenicity of a combined quadrivalent influenza and recombinant SARS-CoV-2 vaccine in Sprague-Dawley rats for both primary and booster immunization

**DOI:** 10.3389/fimmu.2026.1849279

**Published:** 2026-07-17

**Authors:** Jinjin Shao, Chengda Zhang, Guangbiao She, Tian Qin, Jinyao Zhang, Minxi Fang, Dan Zhao, Linying Wang, Lijuan Xia, Qian Yang, Lili Zhang, Yanling Zhang, Siming Zhang, Jiahong Wang, Zhiqi Xie, Yunxiang Chen, Ying Chen, Enqi Huang, Lijiang Zhang

**Affiliations:** 1 Zhejiang Key Laboratory of High-level Biosafety and Biomedical Transformation, Hangzhou Medical College, Hangzhou, China; 2 Key Laboratory of Drug Safety Evaluation and Research of Zhejiang Province, Center of Safety Evaluation and Research, Hangzhou Medical College, Hangzhou, China; 3 Engineering Research Center of Novel Vaccine of Zhejiang Province, Hangzhou Medical College, Hangzhou, China; 4 Anhui Zhifei Longcom Biopharmaceutical Co., Ltd., Hefei, China; 5 Recombinant Vaccine Research and Development Joint Laboratory of Anhui Province, Hefei, China; 6 Wuyi First People’s Hospital, Affiliated Hospital, School of Medicine, Hangzhou City University, Hangzhou, China; 7 School of Public Health, Hangzhou Medical College, Hangzhou, China

**Keywords:** booster vaccination, combined vaccine, immunogenicity, influenza virus, safety, SARS-CoV-2

## Abstract

**Introduction:**

The concurrent circulation of influenza viruses and SARS‑CoV‑2 continues to strain global public health systems. Vaccination remains the cornerstone of defense, and combined vaccines offer a strategic advantage by simplifying logistics, reducing costs, and improving coverage.

**Methods:**

In this set of studies, we evaluated the safety and immunogenicity of a novel combined quadrivalent influenza and recombinant COVID‑19 vaccine (developed by Anhui Zhifei Longcom Biopharmaceutical Co., Ltd.), known as the Flu‑CoV2 vaccine, in Sprague‑Dawley rats. Three independent studies were conducted in accordance with ICH S6(R1) guidelines for Preclinical Safety Evaluation of Biotechnology‑Derived Pharmaceuticals: primary immunization (Study 1) and booster immunization following a prior history of either influenza (Study 2) or COVID‑19 (Study 3) vaccination. Immunogenicity was determined by immunoglobulin G (IgG) antibody enzyme‑linked immunosorbent assay, plaque reduction neutralization tests for SARS‑CoV‑2, and hemagglutination inhibition assays for influenza viruses.

**Results:**

No severe, life‑threatening, or fatal adverse reactions occurred during any of the studies. Transient injection‑site nodules in adjuvanted groups resolved completely, and all transient post‑vaccination shifts in hematologic and biochemical parameters normalized by the end of the recovery period. As a primary regimen, the Flu‑CoV2 vaccine effectively induced strong IgG and neutralizing antibody responses against both influenza and SARS‑CoV‑2. Administered as a booster, it markedly augmented specific antibody levels in pre‑immune models, demonstrating its potential to broadly enhance recall immune responses.

**Conclusions:**

These preclinical data demonstrate a favorable safety profile and potent immunogenicity, supporting the further development of the Flu‑CoV2 vaccine as a promising tool to address the dual threat of influenza and COVID‑19.

## Introduction

The concurrent prevalence of influenza and SARS-CoV-2 is an ongoing global public health challenge. According to World Health Organization (WHO) estimates, approximately 1 billion cases of seasonal influenza occur annually, resulting in 3 to 5 million severe illnesses and 290,000 to 650,000 respiratory-related deaths (1). COVID-19 has caused hundreds of millions of confirmed cases worldwide since its emergence at the end of 2019 (2). The United States, as the country with the highest number of confirmed cases and deaths, has reported over 400,000 cumulative COVID-19-related deaths to date (3, 4). Vaccination remains the most effective strategy to mitigate these threats, eliciting specific immune responses that substantially reduce the risk of severe illness and mortality following infection (5, 6). WHO data confirm that COVID-19 vaccines provide robust protection against infection, severe disease, and death, with fully vaccinated individuals having significantly lower risk of hospitalization and death (7, 8). Similarly, US Centers for Disease Control and Prevention (CDC) reports highlight the benefits of influenza vaccination in reducing influenza-related hospitalizations and deaths, with varying effectiveness in different settings in the 2024–2025 season, but with overall protective efficacy (9). However, the need for annual vaccination, driven by viral antigenic drift and waning antibody immunity, places a recurring burden on individuals and public health systems (10, 11).

In this context, combination vaccines are urgently needed. Combination vaccines integrate antigenic components from two or more pathogens into a single formulation, and include multivalent vaccines targeting different serotypes/subtypes of the same pathogen and multicomponent vaccines targeting multiple distinct pathogens (12). Such vaccines offer considerable advantages: They reduce the number of injections required, decrease logistical costs, improve immunization coverage, minimize adverse events associated with multiple vaccinations, and can elicit synergistic or complementary immune responses against different infectious diseases (13–17). Currently, multiple initiatives are underway worldwide to develop combined influenza-SARS-CoV-2 vaccines using innovative platforms, including mRNA technology, recombinant protein approaches, virus-like particles, and nanoparticle designs (18–23). Although no such combination vaccine has yet received regulatory approval, several candidate vaccines have advanced to clinical trials with promising interim results, laying a foundation for future implementation (24, 25).

Among the various platforms under development, recombinant protein subunit vaccines offer distinct advantages for combined formulations. mRNA vaccines provide strong immunogenicity and flexible design but require ultracold-chain storage and are associated with higher reactogenicity, which may limit their deployment in resource-limited settings (26, 27). Inactivated virus vaccines offer broad antigenic coverage but rely on live virus culture, require multiple doses, and elicit relatively weaker immunogenicity (28). In contrast, recombinant protein vaccines are stable at 2–8 °C, fully compatible with existing influenza vaccine cold-chain infrastructure, and have a well-documented safety profile in elderly and immunocompromised populations due to their inherently low reactogenicity (27, 29). These characteristics make the recombinant protein platform a practical and scalable approach for developing combined influenza–COVID-19 vaccines.

Anhui Zhifei Longcom Biopharmaceutical in China has developed a combined quadrivalent influenza and recombinant SARS-CoV-2 vaccine. This candidate vaccine incorporates hemagglutinin (HA) proteins from four influenza strains (H1N1, H3N2, B/Yamagata, and B/Victoria) along with a recombinant fusion antigen consisting of the nucleocapsid protein (N) and receptor-binding domain (RBD) of SARS-CoV-2. In this study, we evaluated this combined vaccine in Sprague-Dawley (SD) rats through repeated intramuscular injections. We assessed its safety profile and immunogenicity as a primary immunization series, identifying potential target organs or tissues susceptible to toxicity on repeated vaccine administration. Furthermore, we examined its booster immunogenicity effects in rats pre-immunized with either a SARS-CoV-2 (CoV2) or influenza (Flu) vaccine, characterizing immune interactions to inform clinical trial design and highlight key parameters for future clinical monitoring.

## Results

### General observations, body weight, and food consumption

**Figure 1:**
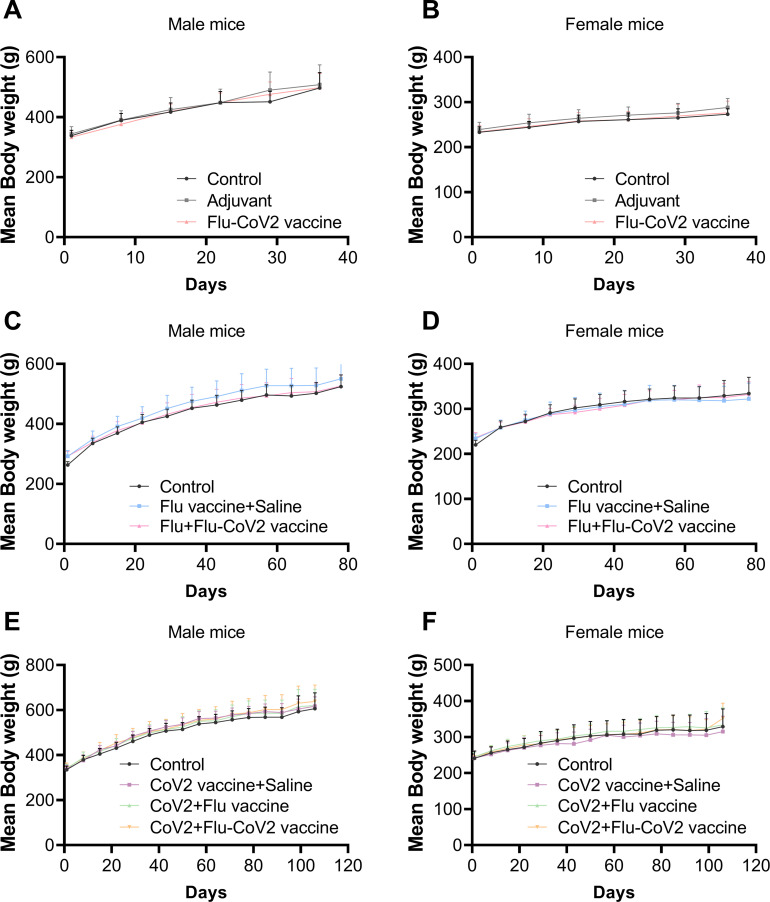
Body weight after repeated doses of flu-CoV2 vaccine. **(A)** Body weight of male and **(B)** female rats in Study 1. **(C)** Body weight of male and **(D)** female rats in Study 2. **(E)** Body weight of male and **(F)** female rats in Study 3. Data are expressed as mean ± SD and groups were compared using one-way ANOVA. The examination of drug withdrawal used 10 male and 10 female rats per group. The examination at the end of the recovery period used 5 male and 5 female rats per group. Study 1, primary immunization with the of Flu-CoV2 vaccine; Study 2, booster immunization with the Flu-CoV2 vaccine in rats with prior influenza immunization; Study 3, booster immunization with the Flu-CoV2 in rats with prior SARS-CoV-2 immunization.

**Figure 2:**
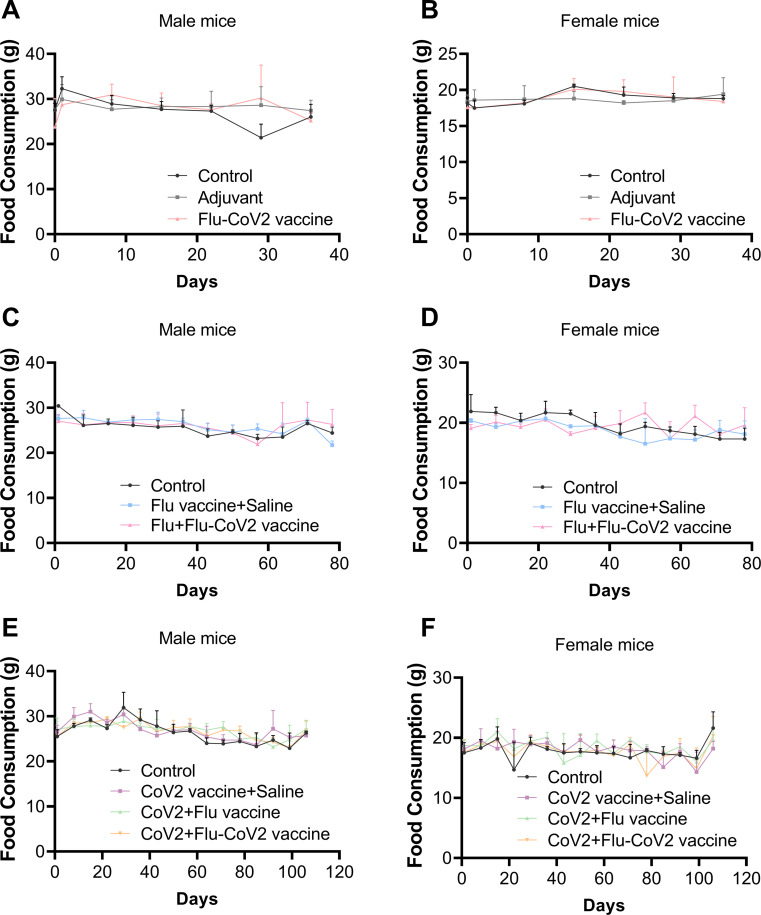
Food consumption after repeated doses of flu-CoV2 vaccine. **(A)** Food consumption of male and **(B)** female rats in Study 1. **(C)** Food consumption of male and **(D)** female rats in Study 2. **(E)** Food consumption of male and **(F)** female rats in Study 3. Data are expressed as mean ± SD and groups were compared using one-way ANOVA. The examination of drug withdrawal used 10 male and 10 female rats per group. The examination at the end of the recovery period used 5 male and 5 female rats per group. Study 1, primary immunization with the of Flu-CoV2 vaccine; Study 2, booster immunization with the Flu-CoV2 vaccine in rats with prior influenza immunization; Study 3, booster immunization with the Flu-CoV2 in rats with prior SARS-CoV-2 immunization.

Throughout the study period, palpable nodules at the injection site were observed in rats across the adjuvant, CoV2 vaccine, and Flu-CoV2 vaccine groups. The timing and incidence of these nodules were consistent across groups and were attributed to the intramuscular administration of aluminum-containing adjuvants, as corroborated by histopathological findings. Apart from these localized reactions, no mortality or other overt clinical abnormalities were observed in any animals receiving the combined Flu-CoV2 vaccine, either as a primary or booster immunization. Furthermore, administration of the Flu-CoV2 vaccine did not adversely affect body weight gain or food consumption in either male or female rats. All groups exhibited steady weight gain over time with consistent trends across treatments (**Figure 1**). Food intake was comparable among groups, with only sporadic fluctuations observed at isolated time points (**Figure 2**).

### Hematology and serum biochemistry

In Study 1 (primary immunization), increases in neutrophils (NEUT) and fibrinogen (Fbg) were noted in the Flu-CoV2 vaccine group and adjuvant group (**Table 1**). In Study 2, an increase in eosinophils (EOS) was observed in the Flu vaccine prime combined with Flu-CoV2 vaccine boost group compared with the control group and the Flu vaccine group (**Table 2**). In Study 3, the CoV2 vaccine prime combined Flu-CoV2 vaccine boost group exhibited elevations in leukocyte-related parameters (NEUT, EOS) compared with the control, CoV2 vaccine, and Flu-CoV2 vaccine groups. The CoV2 vaccine prime combined Flu-CoV2 vaccine boost group also displayed transient coagulopathy characterized by shortened prothrombin time, activated partial thromboplastin time, and thrombin time, and decreased Fbg (**Table 3**). All these hematologic alterations returned to normal by the end of the recovery period. The observed changes are likely related to inflammatory and immune responses triggered by vaccination and should be monitored in future clinical studies.

**Table 1:** The hematology analysis of SD rats treated with flu-CoV2 vaccine in study 1.

Parameter	D31									D43								
	Control			Adjuvant			Flu-CoV2 vaccine			Control			Adjuvant			Flu-CoV2 vaccine		
Number of animals	20			20			20			10			10			10		
WBC (10e3/μL)	4.48	±	1.70	4.74	±	2.03	5.45	±	1.65	5.34	±	2.30	5.11	±	2.32	3.96	±	1.40
#NEUT (10e3/μL)	0.53	±	0.18	0.69	±	0.30	1.10	±	0.53**^△^	0.78	±	0.43	0.75	±	0.28	0.78	±	0.27
#LYMPH (10e3/μL)	3.77	±	1.63	3.83	±	1.79	4.11	±	1.37	4.36	±	1.85	4.14	±	1.99	3.02	±	1.14
#MONO (10e3/μL)	0.07	±	0.03	0.10	±	0.05	0.10	±	0.05	0.08	±	0.05	0.09	±	0.06	0.06	±	0.04
#EOS (10e3/μL)	0.06	±	0.02	0.07	±	0.03	0.07	±	0.03	0.08	±	0.05	0.07	±	0.02	0.07	±	0.04
#BASO (10e3/μL)	0.01	±	0.01	0.01	±	0.01	0.01	±	0.01	0.01	±	0.01	0.01	±	0.01	0.00	±	0.01
#LUC (10e3/μL)	0.04	±	0.02	0.04	±	0.03	0.05	±	0.03	0.04	±	0.02	0.04	±	0.02	0.02	±	0.01
%NEUT (%)	13.0	±	5.1	15.4	±	5.4	20.6	±	6.8**^△△^	14.5	±	5.2	15.6	±	3.4	20.4	±	4.8**^△^
%LYMPH (%)	82.8	±	6.1	80.2	±	5.4	75.2	±	7.0**^△^	81.8	±	5.2	80.1	±	3.8	75.6	±	5.3**^△^
%MONO (%)	1.6	±	0.6	2.0	±	0.6	1.8	±	0.6	1.5	±	0.5	1.8	±	0.7	1.5	±	0.7
%EOS (%)	1.6	±	0.9	1.4	±	0.5	1.4	±	0.6	1.4	±	0.5	1.7	±	0.6	1.9	±	1.1
%BASO (%)	0.2	±	0.1	0.2	±	0.1	0.2	±	0.1	0.09	±	0.06	0.18	±	0.06**	0.11	±	0.09*
%LUC (%)	0.9	±	0.3	0.8	±	0.3	0.9	±	0.3	0.7	±	0.3	0.7	±	0.3	0.5	±	0.2
RBC (10e6/μL)	7.55	±	0.70	7.28	±	0.58	7.26	±	0.51	7.70	±	0.47	7.55	±	0.40	7.35	±	0.27
HGB (g/L)	145	±	14	140	±	10	139	±	10	144	±	8	143	±	4	139	±	6
HCT (%)	42.9	±	4.2	41.9	±	3.1	42.1	±	2.9	42.8	±	1.9	43.0	±	1.4	41.9	±	1.6
MCV (fL)	56.8	±	1.5	57.6	±	1.8	57.9	±	1.2	55.7	±	1.5	57.0	±	1.5	57.1	±	1.4
MCH (Pg)	19.2	±	0.5	19.2	±	0.8	19.1	±	0.6	18.7	±	0.5	19.0	±	0.6	18.8	±	0.6
MCHC (g/dL)	338	±	5	333	±	6*	330	±	6**	336	±	5	333	±	7	330	±	7
RDW (%)	11.1	±	0.4	11.1	±	0.4	11.6	±	0.5**^△△^	11.7	±	0.4	12.0	±	0.6	12.4	±	0.9
PLT (10e3/μL)	1012	±	90	1040	±	113	1039	±	61	1046	±	82	1036	±	110	1056	±	150
MPV (fL)	9.3	±	0.8	9.8	±	0.9	10.6	±	0.9**^△△^	9.5	±	0.6	10.2	±	0.7*	10.5	±	0.8**
%RETIC (%)	2.22	±	0.48	2.41	±	0.42	2.98	±	0.76**^△^	2.10	±	0.50	2.51	±	0.60	2.58	±	0.54
#RETIC (10e9/L)	165.9	±	32.6	174.6	±	27.2	214.5	±	46.8**^△△^	161.2	±	36.9	189.1	±	44.9	189.1	±	38.7
PT (s)	9.0	±	1.4	8.6	±	0.9	8.5	±	0.7	8.4	±	0.8	8.5	±	0.7	8.3	±	0.5
Fbg (g/L)	1.592	±	0.311	1.880	±	0.383*	2.410	±	0.523**^△△^	1.651	±	0.351	1.597	±	0.292	1.582	±	0.358
APTT (s)	19.9	±	2.1	19.1	±	1.6	18.9	±	1.3	18.6	±	1.9	17.6	±	0.9	18.3	±	0.8
TT (s)	67.3	±	22.1	60.2	±	16.0	57.7	±	12.7	61.4	±	26.6	53.3	±	14.9	59.9	±	17.4

The data were expressed as mean ± SD. *P<0.05, **P<0.01 vs control group; ^△^P<0.05, ^△△^P<0.01 vs adjuvant group. WBC, white blood cell; RBC, red blood cell count; HGB, hemoglobin concentration; HCT, hematocrit; MCV, mean cell volume; MCH, mean cell hemoglobin; MCHC, mean cell hemoglobin concentration; RDW, Red blood Cell distribution width; PLT, platelets; MPV, Mean platelet volume; NEUT, neutrophiles; LYMPH, lymphocytes; MONO, monocytes; EOS, eosinophils; BASO, basophiles; LUC, large unstained cells; RETIC, reticulocyte; PT, Prothrombin time; Fbg, fibrinogen; APTT, activated partial thromboplastin time; TT, trombin time.

**Table 2:** The hematology analysis of SD rats treated with flu-CoV2 vaccine in study 2.

Parameter	D74									D85								
	Control			Flu vaccine+Saline			Flu+Flu-CoV2 vaccine			Control			Flu vaccine+Saline			Flu+Flu-CoV2 vaccine		
Number of animals	20			20			20			10			10			10		
WBC (10e3/μL)	3.86	±	2.16	3.45	±	1.45	4.48	±	2.18	5.90	±	1.81	4.87	±	1.61	5.39	±	2.68
#NEUT (10e3/μL)	0.49	±	0.31	0.49	±	0.31	0.71	±	0.40	0.68	±	0.25	0.56	±	0.22	0.64	±	0.25
#LYMPH (10e3/μL)	3.22	±	1.87	2.83	±	1.16	3.57	±	1.81	4.93	±	1.60	4.09	±	1.38	4.47	±	2.38
#MONO (10e3/μL)	0.07	±	0.04	0.06	±	0.04	0.08	±	0.03	0.13	±	0.06	0.10	±	0.05	0.14	±	0.07
#EOS (10e3/μL)	0.06	±	0.03	0.05	±	0.02	0.08	±	0.04*▴▴	0.08	±	0.03	0.07	±	0.03	0.08	±	0.03
#BASO (10e3/μL)	0.00	±	0.01	0.00	±	0.00	0.01	±	0.01	0.01	±	0.01	0.01	±	0.01	0.01	±	0.01
#LUC (10e3/μL)	0.02	±	0.02	0.02	±	0.01	0.03	±	0.02	0.06	±	0.03	0.04	±	0.02	0.05	±	0.04
%NEUT (%)	12.9	±	4.5	13.7	±	4.7	16.1	±	5.4	11.5	±	3.1	11.8	±	4.2	12.7	±	3.1
%LYMPH (%)	82.8	±	4.8	82.3	±	4.9	79.3	±	5.4	83.5	±	3.4	83.4	±	5.0	81.9	±	4.2
%MONO (%)	1.8	±	0.8	1.7	±	0.6	1.9	±	0.8	2.3	±	0.9	2.2	±	0.7	2.8	±	1.0
%EOS (%)	1.7	±	0.6	1.4	±	0.5	1.9	±	0.9	1.4	±	0.5	1.5	±	0.5	1.7	±	0.5
%BASO (%)	0.1	±	0.1	0.1	±	0.1	0.2	±	0.1	0.2	±	0.1	0.2	±	0.1	0.2	±	0.1
%LUC (%)	0.6	±	0.3	0.6	±	0.3	0.6	±	0.2	1.0	±	0.5	0.9	±	0.3	0.8	±	0.5
RBC (10e6/μL)	7.71	±	0.62	7.67	±	0.61	7.60	±	0.53	7.70	±	0.65	7.57	±	0.68	7.81	±	0.66
HGB (g/L)	141	±	8	141	±	9	139	±	8	139	±	7	136	±	5	139	±	7
HCT (%)	44.3	±	2.7	44.2	±	2.6	44.0	±	2.3	43.0	±	2.8	41.9	±	2.6	43.1	±	2.7
MCV (fL)	57.6	±	2.1	57.7	±	2.0	57.9	±	2.0	55.9	±	1.7	55.5	±	2.3	55.3	±	2.0
MCH (Pg)	18.3	±	1.0	18.4	±	0.8	18.3	±	0.8	18.1	±	0.7	18.1	±	1.0	17.9	±	0.9
MCHC (g/dL)	318	±	9	318	±	7	316	±	8	325	±	7	326	±	8	323	±	8
RDW (%)	12.0	±	0.7	12.1	±	0.7	11.8	±	0.6	12.3	±	0.8	12.1	±	1.0	12.4	±	0.6
PLT (10e3/μL)	1070	±	112	1026	±	97	1061	±	74	952	±	113	966	±	117	904	±	112
MPV (fL)	9.5	±	0.7	9.7	±	0.7	9.7	±	0.5	7.7	±	0.5	8.3	±	0.7*	8.6	±	0.6**
%RETIC (%)	2.30	±	0.45	2.50	±	0.64	2.21	±	0.58	2.27	±	0.37	2.24	±	0.43	2.39	±	0.41
#RETIC (10e9/L)	176.2	±	29.5	189.9	±	41.2	166.4	±	37.3	174.7	±	32.6	168.1	±	28.6	185.0	±	24.0
PT (s)	8.9	±	1.2	8.4	±	0.8	8.5	±	1.0	8.7	±	1.0	8.7	±	0.9	8.7	±	1.1
Fbg (g/L)	1.688	±	0.295	1.950	±	0.578	1.852	±	0.302	1.658	±	0.352	1.631	±	0.391	1.664	±	0.308
APTT (s)	18.0	±	2.6	16.4	±	2.3	17.5	±	2.3	18.9	±	2.3	19.4	±	1.7	18.9	±	2.2
TT (s)	102.06	±	17.88	104.41	±	14.79	99.57	±	15.35	62.67	±	26.69	57.34	±	17.62	60.15	±	17.71

The data were expressed as mean ± SD. *P<0.05, **P<0.01 vs control group; ^▴▴^P<0.01 vs Flu vaccine group. WBC, white blood cell; RBC, red blood cell count; HGB, hemoglobin concentration; HCT, hematocrit; MCV, mean cell volume; MCH, mean cell hemoglobin; MCHC, mean cell hemoglobin concentration; RDW, Red blood Cell distribution width; PLT, platelets; MPV, Mean platelet volume; NEUT, neutrophiles; LYMPH, lymphocytes; MONO, monocytes; EOS, eosinophils; BASO, basophiles; LUC, large unstained cells; RETIC, reticulocyte; PT, Prothrombin time; Fbg, fibrinogen; APTT, activated partial thromboplastin time; TT, trombin time.

**Table 3:** The hematology analysis of SD rats treated with flu-CoV2 vaccine in study 3.

Parameter	D101												D113											
	Control			CoV2 vaccine+Saline			CoV2+ Flu vaccine			CoV2+ Flu-CoV2 vaccine			Control			CoV2 vaccine+Saline			CoV2+ Flu vaccine			CoV2+ Flu-CoV2 vaccine		
Number of animals	20			20			20			20			10			10			10			10		
WBC (10e3/μL)	4.57	±	1.60	3.67	±	1.35	3.91	±	1.70	4.43	±	1.93	5.29	±	2.85	4.73	±	2.06	4.92	±	2.64	4.76	±	2.23
#NEUT (10e3/μL)	0.75	±	0.40	0.61	±	0.26	0.65	±	0.38	0.94	±	0.42▴▴▪	0.77	±	0.40	0.78	±	0.21	0.73	±	0.37	0.68	±	0.28
#LYMPH (10e3/μL)	3.60	±	1.35	2.86	±	1.10	3.06	±	1.40	3.24	±	1.56	4.28	±	2.39	3.73	±	1.94	3.95	±	2.20	3.83	±	1.94
#MONO (10e3/μL)	0.10	±	0.04	0.10	±	0.05	0.10	±	0.06	0.10	±	0.04	0.14	±	0.08	0.10	±	0.05	0.12	±	0.05	0.12	±	0.06
#EOS (10e3/μL)	0.08	±	0.03	0.07	±	0.03	0.07	±	0.03	0.11	±	0.04**▴▴▪▪	0.07	±	0.04	0.08	±	0.03	0.09	±	0.04	0.09	±	0.03
#BASO (10e3/μL)	0.01	±	0.01	0.00	±	0.01	0.00	±	0.00	0.01	±	0.01	0.01	±	0.01	0.00	±	0.01	0.01	±	0.01	0.01	±	0.01
#LUC (10e3/μL)	0.03	±	0.01	0.03	±	0.01	0.03	±	0.02	0.04	±	0.02	0.03	±	0.02	0.03	±	0.02	0.03	±	0.02	0.04	±	0.02
%NEUT (%)	16.5	±	7.2	16.9	±	4.7	16.6	±	5.3	21.6	±	6.0**▴▪▪	14.9	±	3.3	18.8	±	7.4	15.3	±	2.8	15.1	±	5.3
%LYMPH (%)	78.7	±	7.5	77.8	±	5.1	78.0	±	5.4	71.9	±	7.5**▴▴▪▪	80.4	±	3.5	76.3	±	8.2	79.3	±	3.3	79.2	±	5.3
%MONO (%)	2.2	±	0.8	2.6	±	1.0	2.6	±	1.3	2.6	±	1.1	2.6	±	0.7	2.2	±	1.0	2.6	±	0.5	2.5	±	0.5
%EOS (%)	1.8	±	0.4	1.8	±	0.5	1.9	±	0.7	2.7	±	1.2*▴	1.5	±	0.6	2.0	±	0.9	2.0	±	0.8	2.2	±	1.1
%BASO (%)	0.1	±	0.1	0.1	±	0.1	0.1	±	0.1	0.2	±	0.1	0.1	±	0.1	0.2	±	0.1	0.2	±	0.1	0.1	±	0.1
%LUC (%)	0.8	±	0.3	0.8	±	0.5	0.8	±	0.6	0.9	±	0.5	0.6	±	0.2	0.6	±	0.3	0.7	±	0.4	0.8	±	0.4
RBC (10e6/μL)	7.92	±	0.62	7.82	±	0.75	7.68	±	0.77	7.59	±	0.58	7.75	±	0.78	7.55	±	0.54	7.67	±	0.76	7.66	±	0.80
HGB (g/L)	142	±	8	141	±	8	139	±	6	136	±	5	137	±	8	133	±	4	139	±	9	135	±	6
HCT (%)	43.1	±	2.6	43.2	±	2.9	42.4	±	2.3	41.8	±	2.1	43.1	±	3.0	42.1	±	1.6	43.1	±	3.5	42.4	±	2.4
MCV (fL)	54.6	±	2.0	55.4	±	2.4	55.5	±	2.8	55.3	±	2.1	55.7	±	2.2	56.0	±	2.7	56.3	±	2.4	55.6	±	3.3
MCH (Pg)	18.0	±	0.8	18.1	±	0.9	18.2	±	1.2	18.0	±	0.9	17.8	±	1.1	17.7	±	1.1	18.2	±	1.0	17.8	±	1.4
MCHC (g/dL)	329	±	8	326	±	6	328	±	5	326	±	7	319	±	7	316	±	6	322	±	7	319	±	8
RDW (%)	11.9	±	0.9	11.6	±	0.9	11.7	±	0.9	11.7	±	0.6	12.6	±	1.2	12.8	±	1.2	12.4	±	1.1	12.4	±	1.4
PLT (10e3/μL)	980	±	111	966	±	104	959	±	77	940	±	87	868	±	107	916	±	114	927	±	99	958	±	164
MPV (fL)	8.2	±	0.5	9.0	±	0.5**	9.2	±	0.5**	8.9	±	0.6**	8.2	±	0.5	8.6	±	0.5	8.4	±	0.4	8.1	±	0.7
%RETIC (%)	1.94	±	0.37	1.75	±	0.43	1.86	±	0.26	1.79	±	0.43	2.18	±	0.41	2.38	±	0.53	2.22	±	0.25	2.45	±	0.56
#RETIC (10e9/L)	152.3	±	26.5	135.2	±	30.2	142.8	±	26.1	134.6	±	27.2	167.9	±	32.1	177.7	±	34.8	170.7	±	30.8	188.7	±	55.1
PT (s)	9.5	±	0.6	7.8	±	0.3**	9.2	±	0.7▴▴	7.8	±	0.3**▪▪	9.9	±	1.9	9.9	±	1.7	9.7	±	1.6	9.5	±	1.1
Fbg (g/L)	1.990	±	0.255	1.417	±	0.162**	2.349	±	0.501*▴▴	1.526	±	0.331**▪▪	1.765	±	0.424	1.793	±	0.362	1.757	±	0.473	1.841	±	0.459
APTT (s)	20.6	±	1.0	17.1	±	1.0**	19.4	±	1.1**▴▴	17.6	±	1.4**▪▪	20.8	±	4.4	19.4	±	4.8	19.1	±	4.0	18.8	±	3.2
TT (s)	73.96	±	22.61	51.98	±	14.96**	63.84	±	14.17▴	57.40	±	15.54**	59.01	±	18.91	58.26	±	13.71	58.49	±	14.65	58.21	±	12.64

The data were expressed as mean ± SD. *P<0.05, **P<0.01 vs control group; ^▴^P<0.05, ^▴▴^P<0.01 vs CoV2 vaccine group; ^▪^P<0.05, ^▪▪^P<0.01 vs CoV2+Flu vaccine group. WBC, white blood cell; RBC, red blood cell count; HGB, hemoglobin concentration; HCT, hematocrit; MCV, mean cell volume; MCH, mean cell hemoglobin; MCHC, mean cell hemoglobin concentration; RDW, Red blood Cell distribution width; PLT, platelets; MPV, Mean platelet volume; NEUT, neutrophiles; LYMPH, lymphocytes; MONO, monocytes; EOS, eosinophils; BASO, basophiles; LUC, large unstained cells; RETIC, reticulocyte; PT, Prothrombin time; Fbg, fibrinogen; APTT, activated partial thromboplastin time; TT, trombin time.

In Study 1, a decreased albumin-to-globulin ratio (A/G) was observed in the adjuvant and Flu-CoV2 vaccine groups (**Table 4**). In Study 2, apart from the chloride ions, no significant changes in blood biochemistry were observed (**Table 5**). In Study 3, as in Study 1, the CoV2 vaccine combined Flu-CoV2 vaccine group exhibited a decreased A/G ratio accompanied by increased globulin (GLO) levels (**Table 6**). No other significant differences in the biochemical parameters were detected among groups. These changes reflect an amplification or extension of the intended pharmacological immune activation following vaccination.

**Table 4:** The blood biochemistry analysis of SD rats treated with flu-CoV2 vaccine in study 1.

Parameter	D31									D43								
	Control			Adjuvant			Flu-CoV2 vaccine			Control			Adjuvant			Flu-CoV2 vaccine		
Number of animals	20			20			20			10			10			10		
ALT(IU/L)	31.29	±	5.74	33.55	±	11.51	34.60	±	8.02	45.10	±	5.44	31.75	±	5.74**	34.15	±	7.34**
AST(IU/L)	155.85	±	44.41	151.73	±	45.52	135.24	±	49.49	171.41	±	27.10	143.06	±	38.13	138.32	±	58.10
ALP(IU/L)	84.56	±	45.33	81.94	±	46.32	85.61	±	45.70	71.64	±	29.48	73.77	±	35.08	74.97	±	33.71
TBIL(umol/L)	0.613	±	0.350	0.524	±	0.366	0.363	±	0.231	0.861	±	0.328	0.774	±	0.514	0.820	±	0.303
CK(IU/L)	510.1	±	250.7	452.4	±	241.6	372.9	±	252.8	417.7	±	176.8	444.7	±	225.2	404.1	±	318.3
TP(g/L)	63.59	±	9.55	61.32	±	7.86	60.58	±	5.82	63.89	±	8.17	63.27	±	8.25	62.89	±	7.14
ALB(g/L)	34.59	±	5.66	32.43	±	5.15	30.73	±	4.27	34.86	±	5.42	33.63	±	5.51	33.05	±	4.46
GLO (g/L)	29.00	±	4.03	28.89	±	3.06	29.86	±	1.83	29.02	±	2.88	29.64	±	2.83	29.84	±	2.85
A/G	1.19	±	0.07	1.12	±	0.11*	1.03	±	0.10**^△△^	1.20	±	0.09	1.13	±	0.09	1.11	±	0.07
GLU(mmol/L)	5.480	±	0.726	5.521	±	0.857	5.787	±	0.905	5.546	±	0.861	5.485	±	0.507	5.118	±	0.505
BUN(mmol/L)	5.673	±	1.007	6.000	±	0.841	6.009	±	1.004	6.784	±	1.638	7.008	±	2.382	6.702	±	1.664
Crea(umol/L)	47.26	±	5.20	46.01	±	2.95	45.71	±	3.88	53.40	±	7.66	52.66	±	13.32	49.67	±	5.40
CHO(mmol/L)	1.744	±	0.417	1.740	±	0.379	1.659	±	0.348	1.827	±	0.431	1.779	±	0.265	1.793	±	0.405
TG(mmol/L)	0.287	±	0.071	0.328	±	0.102	0.305	±	0.120	0.269	±	0.052	0.294	±	0.088	0.239	±	0.041
K+(mmol/L)	4.21	±	0.46	4.17	±	0.53	4.10	±	0.42	4.06	±	0.37	4.14	±	0.40	4.16	±	0.62
Na+(mmol/L)	141.9	±	1.8	142.0	±	1.7	143.7	±	1.8**^△△^	136.5	±	3.6	138.9	±	1.8*	141.7	±	1.1**^△^
Cl-(mmol/L)	114.3	±	1.9	114.2	±	1.6	115.4	±	1.6	115.0	±	2.2	116.1	±	1.9	115.8	±	0.4
Ca(mmol/L)	2.36	±	0.14	2.33	±	0.15	2.31	±	0.11	2.32	±	0.18	2.35	±	0.12	2.30	±	0.11

The data were expressed as mean ± SD. *P<0.05, **P<0.01 vs control group; ^△^P<0.05, ^△△^P<0.01 vs adjuvant group. ALT, alanine aminotransferase; AST, aspartate aminotransferase; TP, total protein; ALB, albumin; TBIL, total bilirubin; ALP, alkaline phosphatase; r-GT, r-Glutamyltransferase; GLU, glucose; BUN, Blood urea nitrogen; Crea, Creatinine; CHO, cholesterol; TG, triglyceride; CK, creatine phosphokinase; GLO, Globulin; A/G, albumin/globulin ratio.

**Table 5:** The blood biochemistry analysis of SD rats treated with flu-CoV2 Vaccine in study 2.

Parameter	D74									D85								
	Control			Flu vaccine+Saline			Flu+ Flu-CoV2 vaccine			Control			Flu vaccine+Saline			Flu+ Flu-CoV2 vaccine		
Number of animals	20			20			20			10			10			10		
ALT(IU/L)	31.33	±	6.75	29.43	±	7.29	31.67	±	7.16	39.58	±	6.97	36.42	±	6.87	41.86	±	10.60
AST(IU/L)	156.46	±	30.89	146.98	±	43.04	144.98	±	51.08	114.31	±	18.40	107.66	±	21.12	118.79	±	35.74
ALP(IU/L)	64.58	±	35.51	61.03	±	33.90	56.74	±	27.86	62.37	±	34.42	63.96	±	37.15	58.46	±	28.72
TBIL(umol/L)	0.939	±	0.516	1.041	±	0.344	0.898	±	0.557	1.378	±	0.402	1.251	±	0.416	1.257	±	0.415
CK(IU/L)	436.2	±	169.0	413.7	±	198.6	342.4	±	240.4	314.4	±	119.0	283.2	±	135.8	330.9	±	217.5
TP(g/L)	63.41	±	7.34	63.96	±	8.46	60.75	±	8.31	61.59	±	7.56	61.47	±	6.87	64.11	±	9.19
ALB(g/L)	37.90	±	5.96	38.22	±	6.31	35.22	±	5.70	37.06	±	6.04	36.95	±	5.80	38.00	±	6.66
GLO (g/L)	25.50	±	2.10	25.74	±	2.56	25.53	±	2.91	24.53	±	2.39	24.53	±	1.60	26.12	±	3.00
A/G	1.49	±	0.20	1.48	±	0.16	1.38	±	0.12	1.51	±	0.21	1.50	±	0.19	1.45	±	0.16
GLU(mmol/L)	4.960	±	0.572	4.877	±	0.839	4.888	±	0.718	6.275	±	0.893	6.350	±	0.660	6.209	±	0.544
BUN(mmol/L)	6.757	±	1.102	6.773	±	1.278	6.930	±	1.061	5.913	±	1.353	6.290	±	0.904	6.498	±	0.986
Crea(umol/L)	54.78	±	6.12	53.73	±	6.50	53.73	±	6.30	55.96	±	6.05	58.35	±	6.38	57.37	±	5.90
CHO(mmol/L)	1.786	±	0.362	1.890	±	0.459	1.801	±	0.430	1.836	±	0.385	1.865	±	0.447	1.801	±	0.446
TG(mmol/L)	0.287	±	0.071	0.258	±	0.130	0.275	±	0.097	0.327	±	0.088	0.381	±	0.094	0.496	±	0.294
K+(mmol/L)	4.19	±	0.31	4.17	±	0.31	4.13	±	0.35	4.19	±	0.19	4.16	±	0.30	4.22	±	0.30
Na+(mmol/L)	144.0	±	2.1	143.9	±	1.0	143.8	±	1.8	144.0	±	2.2	144.7	±	1.7	144.6	±	1.4
Cl-(mmol/L)	107.5	±	1.1	107.9	±	1.6	108.4	±	1.1	113.3	±	1.7	113.8	±	1.1	115.3	±	1.6**▴
Ca(mmol/L)	2.35	±	0.13	2.35	±	0.15	2.31	±	0.14	2.36	±	0.15	2.34	±	0.12	2.37	±	0.19

The data were expressed as mean ± SD. **P<0.01 vs control group; ^▴^P<0.05 vs Flu vaccine group. ALT, alanine aminotransferase; AST, aspartate aminotransferase; TP, total protein; ALB, albumin; TBIL, total bilirubin; ALP, alkaline phosphatase; r-GT, r-Glutamyltransferase; GLU, glucose; BUN, Blood urea nitrogen; Crea, Creatinine; CHO, cholesterol; TG, triglyceride; CK, creatine phosphokinase; GLO, Globulin; A/G, albumin/globulin ratio.

**Table 6:** The blood biochemistry analysis of SD rats treated with vlu-CoV2 vaccine in study 3.

Parameter	D101												D113											
	Control			CoV2 vaccine+Saline			CoV2+Flu vaccine			CoV2+Flu-CoV2 vaccine			Control			CoV2 vaccine+Saline			CoV2+Flu vaccine			CoV2+Flu-CoV2 vaccine		
Number of animals	20			20			20			20			10			10			10			10		
ALT(IU/L)	38.53	±	12.92	44.13	±	26.32	42.14	±	25.53	42.52	±	17.42	48.37	±	12.73	54.82	±	22.65	43.39	±	5.89	45.76	±	9.23
AST(IU/L)	152.45	±	59.47	157.33	±	45.38	162.03	±	76.25	133.49	±	36.43	115.34	±	17.59	124.61	±	24.31	116.46	±	24.73	94.82	±	16.26*▴▴▪
ALP(IU/L)	54.94	±	26.81	50.45	±	24.71	52.38	±	30.54	48.42	±	26.57	64.48	±	30.55	66.12	±	30.60	61.90	±	34.10	62.67	±	32.16
TBIL(umol/L)	0.967	±	0.392	1.081	±	0.376	0.860	±	0.366	0.810	±	0.423	0.652	±	0.252	0.788	±	0.488	0.839	±	0.472	0.739	±	0.427
CK(IU/L)	479.2	±	275.1	398.3	±	171.7	364.1	±	169.5	248.7	±	124.4**▴	277.8	±	90.3	247.1	±	104.5	256.7	±	155.8	222.5	±	148.8
TP(g/L)	61.38	±	7.39	62.69	±	7.08	61.77	±	7.09	61.26	±	6.50	62.31	±	6.55	63.07	±	9.53	63.28	±	6.52	67.65	±	9.35
ALB(g/L)	36.83	±	5.68	37.85	±	5.94	36.93	±	5.53	35.31	±	5.54	37.37	±	6.31	37.68	±	6.89	37.81	±	5.92	39.42	±	7.14
GLO (g/L)	24.55	±	2.41	24.85	±	1.55	24.85	±	2.03	25.95	±	1.57	24.94	±	1.29	25.39	±	2.94	25.47	±	1.60	28.22	±	2.62*
A/G	1.50	±	0.19	1.52	±	0.19	1.49	±	0.17	1.36	±	0.19*▴▴▪	1.50	±	0.26	1.48	±	0.15	1.49	±	0.23	1.39	±	0.17
GLU(mmol/L)	5.400	±	0.518	5.150	±	0.500	5.183	±	0.619	5.312	±	0.560	6.994	±	0.868	6.520	±	0.788	6.503	±	1.300	6.772	±	0.477
BUN(mmol/L)	7.045	±	1.380	7.157	±	1.795	7.508	±	1.799	6.758	±	1.319	5.945	±	0.986	6.445	±	0.874	6.966	±	1.719	6.042	±	0.882
Crea(umol/L)	53.78	±	5.95	54.05	±	6.13	55.52	±	6.82	53.02	±	4.66	57.21	±	5.90	58.30	±	6.11	58.58	±	5.99	56.44	±	5.41
CHO(mmol/L)	1.701	±	0.465	1.961	±	0.387	1.999	±	0.416	2.033	±	0.397	1.889	±	0.334	1.856	±	0.370	2.037	±	0.414	2.127	±	0.423
TG(mmol/L)	0.259	±	0.085	0.300	±	0.147	0.294	±	0.075	0.285	±	0.103	0.536	±	0.251	0.416	±	0.187	0.422	±	0.208	0.751	±	0.455
K+(mmol/L)	4.22	±	0.26	4.10	±	0.40	4.02	±	0.34	4.07	±	0.35	4.24	±	0.33	4.15	±	0.43	4.22	±	0.29	4.23	±	0.48
Na+(mmol/L)	143.5	±	2.2	143.8	±	1.7	144.6	±	1.0*	145.2	±	1.7**▴▴	143.1	±	1.7	143.5	±	2.1	142.4	±	2.1	143.0	±	1.5
Cl-(mmol/L)	114.1	±	1.9	113.6	±	1.2	114.0	±	1.3	114.6	±	1.2	105.7	±	1.5	106.8	±	1.5	104.5	±	1.7▴▴	105.3	±	1.3▴
Ca(mmol/L)	2.34	±	0.15	2.35	±	0.12	2.32	±	0.12	2.32	±	0.12	2.32	±	0.13	2.30	±	0.13	2.30	±	0.11	2.38	±	0.15

The data were expressed as mean ± SD. *P<0.05, **P<0.01 vs control group; ^▴^P<0.05, ^▴▴^P<0.01 vs CoV2 vaccine group; ^▪^P<0.05, ^▪▪^P<0.01 vs CoV2+Flu vaccine group. ALT, alanine aminotransferase; AST, aspartate aminotransferase; TP, total protein; ALB, albumin; TBIL, total bilirubin; ALP, alkaline phosphatase; r-GT, r-Glutamyltransferase; GLU, glucose; BUN, Blood urea nitrogen; Crea, Creatinine; CHO, cholesterol; TG, triglyceride; CK, creatine phosphokinase; GLO, Globulin; A/G, albumin/globulin ratio.

### Organ coefficients and urinalysis

In all three studies, the Flu-CoV2 vaccine had no adverse effect on organ weights or organ-to-body weight coefficients in either sex (**Supplementary Tables S1–S6**). All urinalysis parameters remained within normal limits, indicating no detectable adverse effect on kidney function or overall body homeostasis (**Supplementary Tables S7–S12**).

### Necropsy and histopathology

**Figure 3:**
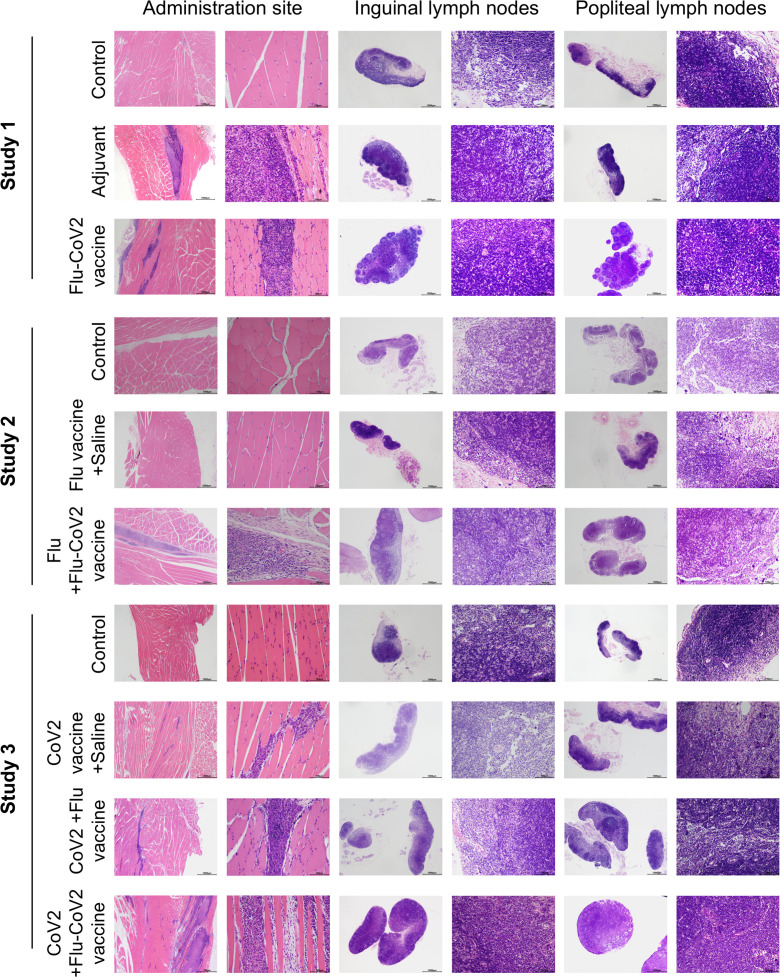
Histopathological results at the injection site and the draining lymph nodes after repeated doses of flu-CoV2 vaccine. Representative images from 20 animals per group. 20×, scale bar = 1000 μm; 200×, scale bar = 100 μm).

A systematic necropsy and histopathological examination were performed on all rats. Consistent with the general observations, rats in all adjuvant-containing groups exhibited subacute inflammatory reactions at the administration site, accompanied by foci of macrophage phagocytosis of the adjuvant. Reactive hyperplasia and enhanced phagocytic activity were also observed in the draining lymph nodes (including the inguinal and popliteal lymph nodes) (**Figure 3**). After a 2-week recovery period, these changes were still present but had almost resolved. Sporadic micro-focal lesions observed in some organs were not dose-related and were considered to be incidental findings unrelated to vaccine administration. Gross and histopathological examinations confirmed the absence of vaccine-related pathological changes in critical organs, such as the heart, liver, and kidneys.

### Immunotoxicity assessment

**Figure 4:**
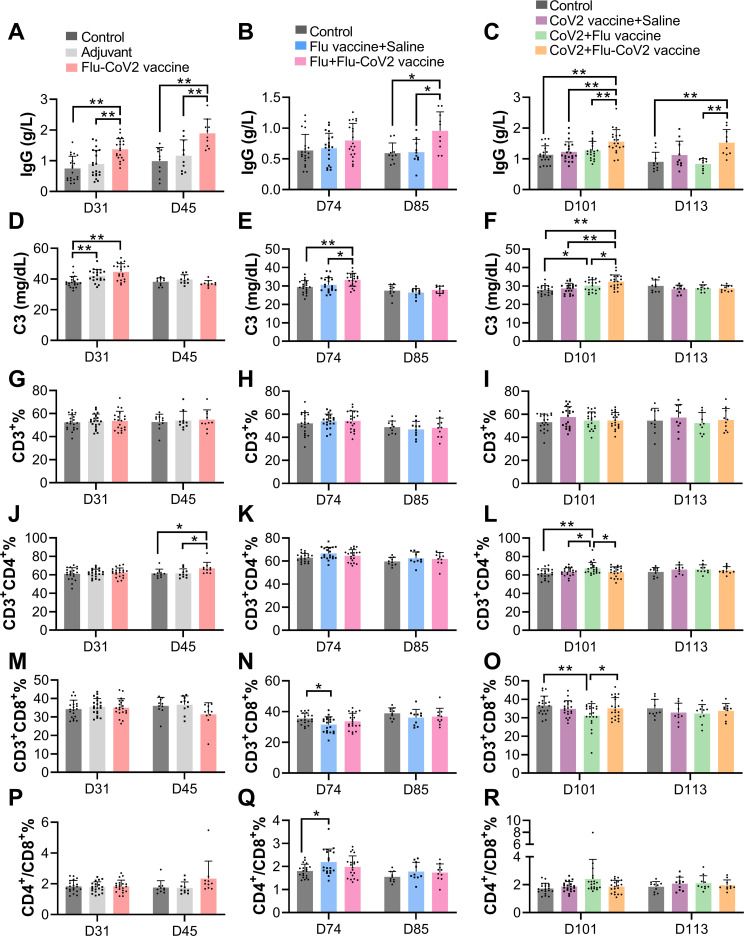
Changes in immunotoxicological indices in rats after repeated doses of flu-CoV2 vaccine. **(A–R)** IgG, C3, CD3^+^, CD3^+^CD4^+^, CD3^+^CD8^+^, and CD4^+^/CD8^+^ levels. Data are expressed as mean ± SD and groups were compared one-way ANOVA. *P< 0.05; **P< 0.01. The examination of drug withdrawal used 10 male and 10 female rats per group. The examination at the end of the recovery period used 5 male and 5 female rats per group.

Analysis of immunotoxicity-related parameters revealed that all groups that received the Flu-CoV2 vaccine showed a significant increase in serum IgG and complement C3 levels immediately after the final dose (**Figures 4A–F**). Elevated IgG levels persisted until the end of the 2-week recovery period, demonstrating a clear time-dependent effect. Cellular immune profiling revealed transient minor fluctuations in T-lymphocyte subsets (CD3^+^, CD4^+^, CD8^+^, and CD4^+^/CD8^+^ ratio), which returned to baseline levels within 2 weeks after immunization (**Figures 4G–I**). Notably, no abnormal changes were observed in the weights or coefficients of immune organs (spleen and thymus) in any group. The alterations in humoral immune parameters are indicative of robust vaccine-induced immune activation rather than toxicity.

### Immunogenicity

**Figure 5:**
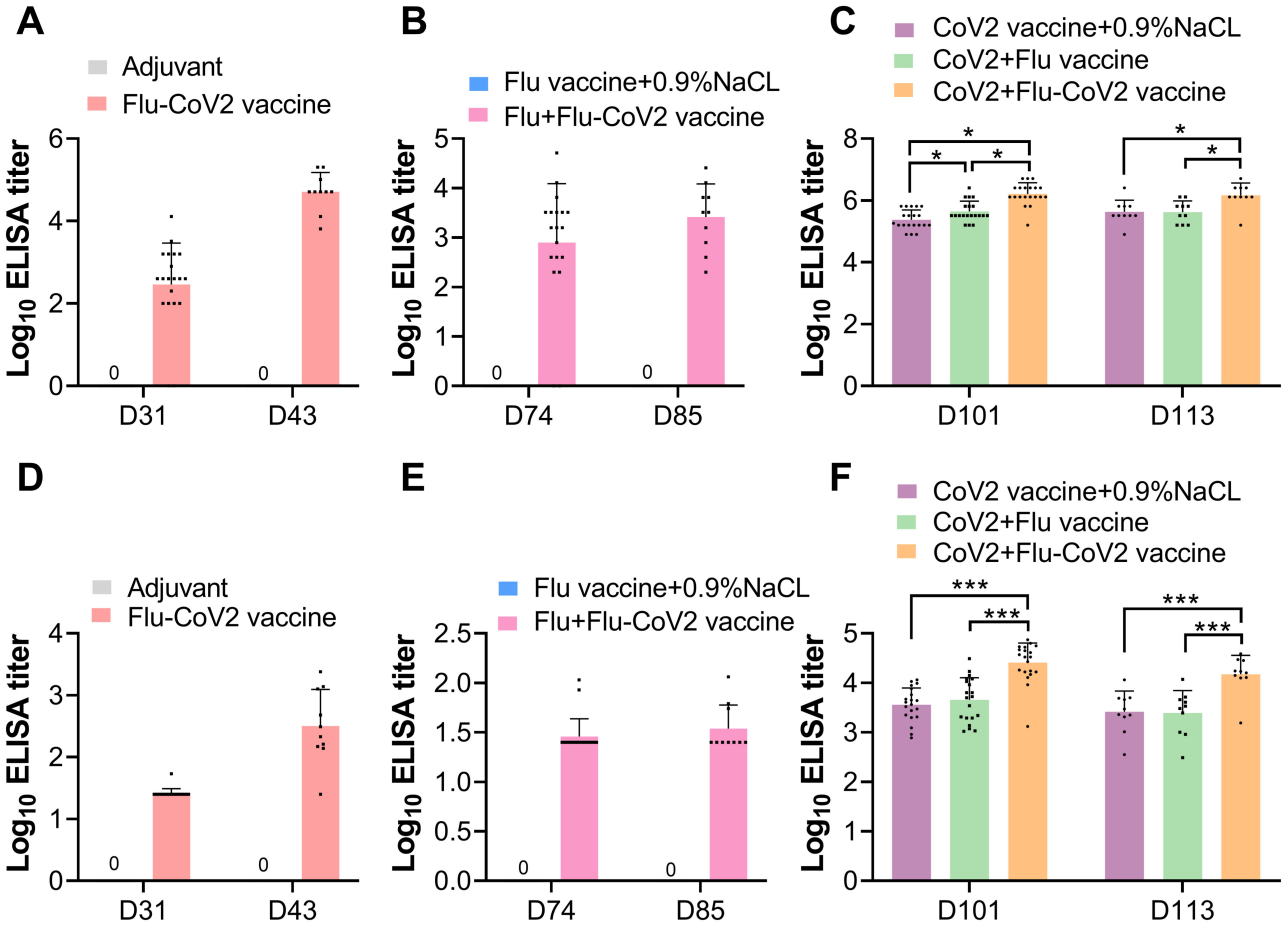
SARS-CoV-2 spike-specific antibody titers following repeated doses of flu-CoV2 vaccine. **(A–C)** Levels of immunoglobulin G antibodies to nucleocapsid protein (NCP) receptor-binding domain (RBD) and **(D–F)** levels of pseudovirus neutralizing antibodies. Antibodies were measured using ELISAs. Data are expressed as mean ± SD and groups were compared using one-way ANOVA. *P< 0.05; ***P< 0.001. The examination of drug withdrawal used 10 male and 10 female rats per group. The examination at the end of the recovery period used 5 male and 5 female rats per group.

**Figure 6:**
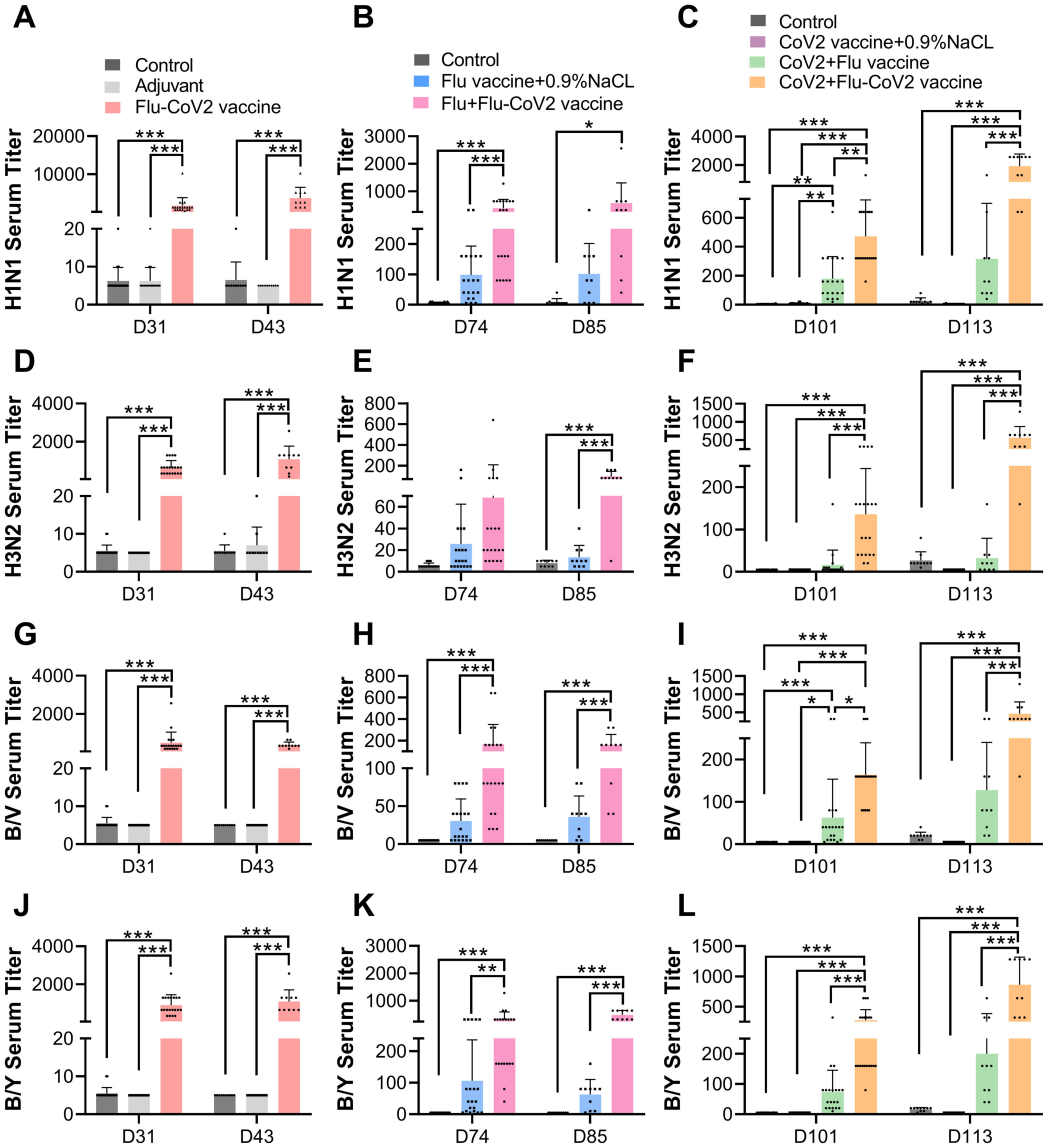
Serum titers against influenza H1N1, H3N2, B/V, and B/Y following repeated doses of Flu-CoV2 vaccine. H1N1 serum titers in **(A)** Study 1; **(B)** Study 2; and **(C)** Study 3. H3N2 serum titers in **(D)** Study 1; **(E)** Study 2; and **(F)** Study 3. B/V serum titers in **(G)** Study 1; **(H)** Study 2; and **(I)** Study 3. B/Y serum titers in **(J)** Study 1; **(K)** Study 2; and **(L)** Study 3. Data are expressed as mean ± SD and groups were compared using one-way ANOVA. *P< 0.05; **P< 0.01; ***P< 0.001. The examination of drug withdrawal used 10 male and 10 female rats per group, The examination at the end of the recovery period used 5 male and 5 female rats per group. H1N1, influenza H1N1; H3N2, influenza H3N2; B/V, influenza B/Victoria; B/Y, influenza B/Yamagata. Study 1, primary immunization with the of Flu-CoV2 vaccine; Study 2, booster immunization with the Flu-CoV2 vaccine in rats with prior influenza immunization; Study 3, booster immunization with the Flu-CoV2 in rats with prior SARS-CoV-2 immunization.

When administered as a primary series, the Flu-CoV2 vaccine induced a strong IgG antibody response against the SARS-CoV-2 nucleocapsid protein (NCP)-RBD antigen and pseudovirus neutralizing antibodies against the SARS-CoV-2 prototype strain. It also elicited strong hemagglutination inhibition (HAI) antibody responses against all four influenza strains (H1N1, H3N2, B/Victoria, and B/Yamagata) (**Figures 5A, D**, **6A, D, G, J**).

In animals primed with a single dose of quadrivalent influenza vaccine (Flu), subsequent booster immunization with the combined Flu-CoV2 vaccine induced strong IgG responses against the SARS-CoV-2 RBD antigen, pseudovirus neutralizing antibodies (prototype strain), and significantly boosted influenza-specific antibody titers. Notably, the SARS-CoV-2 vaccine component within the combined formulation did not interfere with the immunogenicity of the influenza vaccine components but instead significantly enhanced their immunogenicity (**Figures 5B, E**, **6B, E, H, K**).

In rats previously immunized with three doses of CoV2 vaccine, the combined Flu-CoV2 vaccine administered as a booster led to marked elevations in the levels of SARS-CoV-2-specific binding antibodies, pseudovirus neutralizing antibodies (prototype strain), and influenza HAI antibodies. Crucially, the antibody responses against both SARS-CoV-2 and influenza viruses were significantly higher in the group that received the combined Flu-CoV2 booster compared with the group boosted with the Flu vaccine alone (**Figures 5C, F**, **6C, F, I, L**).

## Discussion

Although the development of combination vaccines is an important advancement in immunization strategy, their safety profile is of paramount importance. Previous studies suggest that combination vaccines do not always exhibit the same safety and efficacy as their monovalent counterparts (25, 30). Furthermore, evidence regarding the safety and immunogenicity of combination vaccines used as booster doses in pre-immune populations is limited, contributing to public hesitancy (31). In this study, we comprehensively evaluated a combined quadrivalent influenza and recombinant COVID-19 vaccine, developed by Anhui Zhifei Longcom Biopharmaceutical, not only as a primary immunization but also as a booster in the context of pre-existing immunity against either influenza or SARS-CoV-2.

In all three studies, palpable injection-site nodules were observed in groups receiving formulations containing aluminum adjuvant, including the adjuvant, CoV2 vaccine, and Flu-CoV2 vaccine groups. Aluminum adjuvants are widely used to enhance immunogenicity by increasing antigen surface area and promoting local immune cell recruitment (32, 33). However, they are known to cause transient local reactions, including nodule formation, so this finding is consistent with previous reports (34–36). Our immunotoxicity assessment revealed that the Flu-CoV2 vaccine induced only mild and transient fluctuations in T-cell subsets, with all values returning to baseline within 2 weeks after immunization. Importantly, no vaccine-related systemic adverse reactions or significant changes in immune organ coefficients were detected. These findings indicate that the vaccine has an acceptable local reactogenicity profile and no persistent adverse effects on cellular immunity. Moreover, regardless of prior vaccination history, the combined vaccine did not elicit additional systemic or local toxicity, supporting its favorable safety profile even when administered as a booster after immunization with monovalent vaccines.

In terms of immunogenicity, the Flu-CoV2 vaccine demonstrated a robust ability to elicit and enhance humoral immune responses. As a primary immunization, it induced substantial IgG and neutralizing antibody responses against SARS-CoV-2 and all four influenza strains. When administered as a booster, it significantly elevated pre-existing antibody levels against both SARS-CoV-2 and influenza antigens, exceeding the response observed in groups receiving influenza vaccine alone. Notably, the SARS-CoV-2 component did not interfere with the immunogenicity of influenza antigens but augmented their effect. The sustained increase in serum IgG levels further corroborates its potent immunogenic effect. These results suggest that the Flu-CoV2 vaccine can provide broad and enhanced protection, supporting its potential use in simplifying vaccination schedules and improving coverage without compromising immunogenicity.

Compared with other combined vaccine platforms, the Flu-CoV2 recombinant protein vaccine demonstrated a favorable safety profile. mRNA-based combined vaccines have been associated with injection-site erythema, pyrexia, systemic inflammatory responses, and acute-phase cytokine changes (25, 37), while inactivated virus-based combination vaccines may trigger broader immune complex formation and cytokine release due to their complex antigen composition (30). In the present study, the Flu-CoV2 vaccine elicited only transient, adjuvant-associated local reactions and fully reversible hematological and biochemical changes, with no systemic toxicity across all three studies. A component-dependent difference was observed between the two booster studies: hematological changes were minimal in animals with prior influenza immunity (Study 2) but more pronounced in animals with prior SARS-CoV-2 immunity (Study 3). We attribute this to the larger memory lymphocyte pool generated by the 3-dose CoV2 priming regimen and a potential Th2-biased recall response to the alum-adjuvanted NCP-RBD antigen. All changes were reversible, indicating pharmacological immune activation rather than toxicity.

This study has several limitations. First, Study 1 did not include single-component control groups, which would have enabled direct quantification of additive reactogenicity from component combination. Second, cytokine/chemokine profiling was not performed, limiting characterization of Th1/Th2 polarization. Future studies should incorporate single-component controls and multiplex cytokine assays to address these gaps. Additionally, larger-scale clinical studies are needed to confirm durability of immune responses, monitor rare adverse events, establish correlates of protection, and evaluate responses against emerging variants.

In conclusion, our findings provide robust preclinical evidence supporting the safety and immunogenicity of this combined influenza-SARS-CoV-2 vaccine, both for primary and booster immunization. It offers a promising strategy to mitigate the public health burden of both infections through simplified, effective, and well-tolerated vaccination.

## Materials and methods

### Vaccines

The combined quadrivalent influenza and recombinant SARS-CoV-2 vaccine (hereafter referred to as the “Flu-CoV2 vaccine”) was developed by Anhui Zhifei Longcom Biopharmaceutical Co., Ltd. It is administered as a single intramuscular injection for the prevention of both influenza and COVID-19. The vaccine consists of two components mixed at a 1:1 ratio immediately prior to administration: A quadrivalent inactivated influenza vaccine (split virion, hereafter referred to as the “Flu vaccine”), containing 15 μg hemagglutinin (HA) each of H1N1, H3N2, B/Yamagata (BY), and B/Victoria (BV) strains per 0.5 mL dose (60 μg total HA per 0.5 mL). A recombinant SARS-CoV-2 protein vaccine (Chinese hamster ovary [CHO] cell, hereafter referred to as the “CoV2 vaccine”), was supplied as a 0.5 mL suspension containing 25 μg of NCP-RBD fusion protein and aluminum hydroxide adjuvant (0.25 mg Al per dose). The combined Flu-CoV2 vaccine (1.0 mL/dose) therefore delivers a total protein content of 85 μg (60 μg influenza HA + 25 μg NCP-RBD), with aluminum hydroxide at 0.25 mg Al per dose.

### Animals

Male and female SD rats (7–10 weeks old) were supplied by Zhejiang Vital River Laboratory Animal Technology Co., Ltd. Animals were housed under controlled conditions: temperature 20–26 °C, relative humidity 30–70%, ventilation ≥15 air changes/h, and a 12/12-h light/dark cycle. Lighting was suspended during urine collection. All procedures were approved by the Institutional Animal Care and Use Committee.

### Experimental design

**Figure 7:**
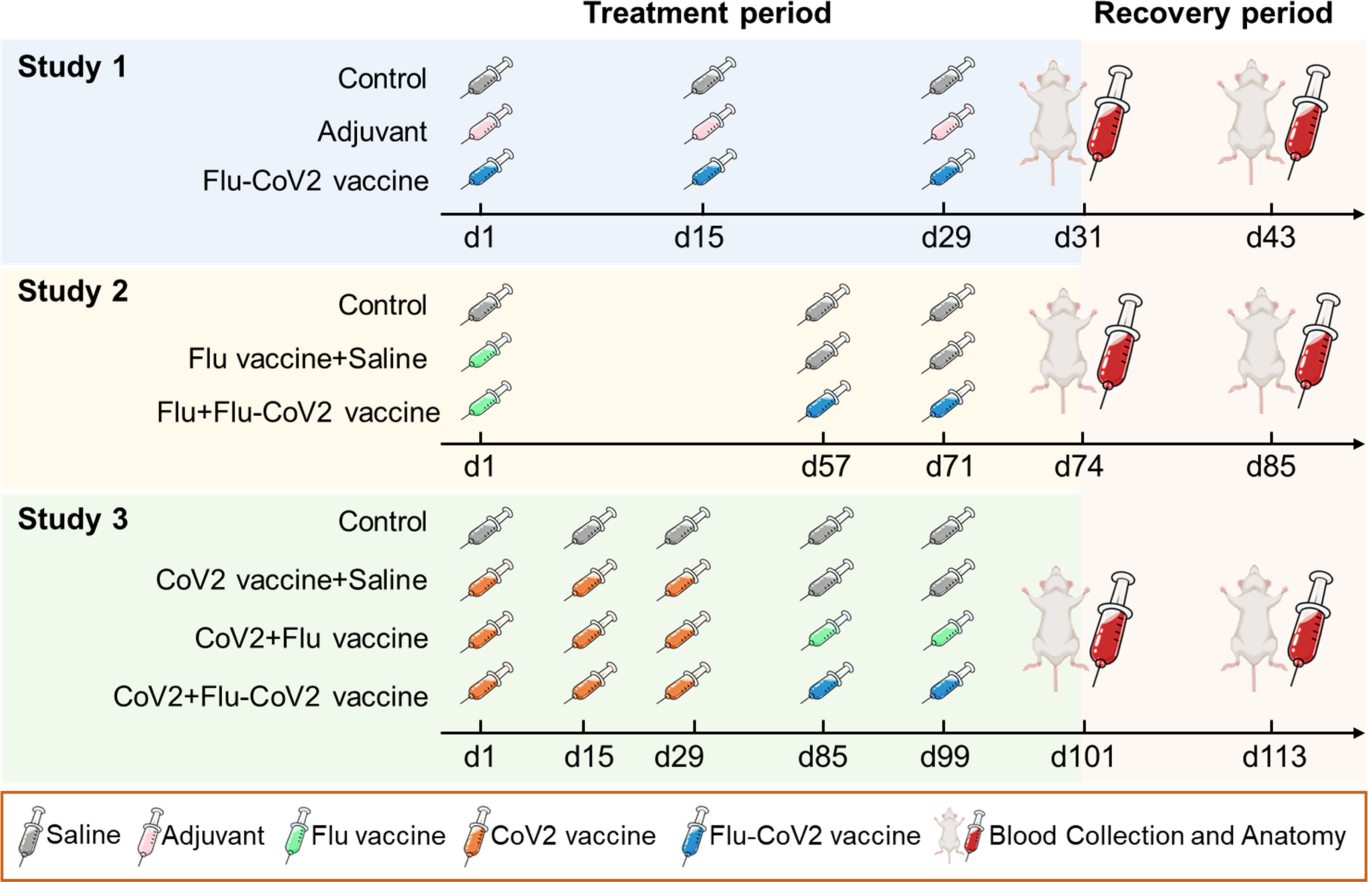
Overview of the study design. Study 1, primary immunization with the of Flu-CoV2 vaccine; Study 2, booster immunization with the Flu-CoV2 vaccine in rats with prior influenza immunization; Study 3, booster immunization with the Flu-CoV2 in rats with prior SARS-CoV-2 immunization.

The study was meticulously planned following the ICH S6(R1) Guidelines for the Preclinical Safety Evaluation of Biotechnology-Derived Pharmaceuticals. The detailed study design is shown in **Figure 7**.

Study 1: Ninety special pathogen free (SPF) SD rats (45 males and 45 females) were randomly assigned by weight to three groups (30 rats per group): control (saline, 1.0 mL/rat), adjuvant (aluminum-adjuvanted COVID-19 vaccine placebo, 0.5 mL/rat), and Flu-CoV2 vaccine (1.0 mL/rat). Immunizations were administered on Days 1, 15, and 29, followed by a 2-week recovery period.

Study 2: Ninety SD rats (45 males and 45 females) were randomly divided into: control (saline 0.5 mL + saline 1.0 mL), Flu vaccine (0.5 mL + saline 1.0 mL), and Flu vaccine + Flu-CoV2 vaccine (0.5 mL + 1.0 mL) groups (30 rats per group). Immunizations were administered on Days 1, 57, and 71, followed by a 2-week recovery period.

Study 3: One hundred twenty SD rats (60 males and 60 females) were allocated to: control (saline 0.5 mL + saline 1.0 mL), CoV2 vaccine (0.5 mL + saline 1.0 mL), CoV2 vaccine + Flu vaccine (0.5 mL + 0.5 mL), and CoV2 vaccine + Flu-CoV2 vaccine (0.5 mL + 1.0 mL) groups (30 rats per group). Immunizations were administered on Days 1, 15, 29, 85, and 99, followed by a 2-week recovery period.

### Clinical examinations

Animals were monitored daily for appearance, behavioral activity, injection-site reactions, glandular secretions, feces/urine characteristics, and mortality. Detailed observations were conducted before and after each vaccine administration. Body weight and food consumption were measured weekly. Food consumption was determined by weighing the amount of feed provided and subtracting the residual feed after 24 h; the difference was divided by the number of animals per cage to obtain the mean daily food intake per animal (g/animal/day).

### Hematology and serum biochemistry

Blood samples were collected under anesthesia (50 mg/kg sodium pentobarbital, administered intraperitoneally) from the abdominal aorta after a 12-h fast. Hematology parameters, coagulation parameters, and serum biochemistry parameters were measured as previously described (38).

### Flow cytometry

EDTA-anticoagulated whole blood (50 μL) was labeled with fluorescent monoclonal antibodies: Anti-RAT CD3 FITC (BD Pharmingen), Anti-RAT CD4 APC (BD Pharmingen), and Anti-RAT CD8a PerCP-Cy5.5 (BD Pharmingen). After 15 min of incubation in the dark, 1 mL of RBC lysis buffer (BD Pharm Lyse) was added and incubated for 15 min. Samples were centrifuged at 1500 rpm for 5 min, the supernatant was discarded, and the pellet was washed once with 1 mL PBS. After a second centrifugation, cells were resuspended in 300 μL PBS and kept in the dark until analysis. T-lymphocyte subsets (CD3^+^, CD4^+^, CD8^+^ percentages and CD4^+^/CD8^+^ ratio) were analyzed using a BD FACS Calibur flow cytometer.

### Immunoglobulin G geometric mean titer assay

SARS-CoV-2 NCP-RBD protein (1000 ng/mL) was coated onto 96-well plates. Serially diluted serum samples were added, followed by horseradish peroxidase (HRP)-conjugated goat anti-rat IgG. TMB substrate was used for color development. Optical density was measured, at 450 nm (OD_450_), and the geometric mean titer (GMT) was calculated as:


$$\text{GMT}={\text{lg}}^{-1}\left(\frac{\sum f.\mathrm{lg}\;X}{\sum f}\right)$$

Where *f* is the number of serum samples and *X* is the reciprocal of the antibody titer.

### Pseudovirus neutralization test

Serum was heat-inactivated (56 °C, 30 min) and centrifuged. Serially diluted serum was mixed with pseudovirus (650 × 50% tissue culture infectious dose [TCID_50_]) and incubated for 1 h. Huh-7 cells were added and cultured for 20–28 h. Luciferase activity was measured, and neutralization inhibition rate was calculated. The half-maximal inhibitory concentration (IC_50_) was determined using the Reed–Muench method.

### Hemagglutination inhibition assay

Serum was treated with receptor-destroying enzyme and adsorbed with chicken red blood cells (RBCs). Antigen was back-titrated to confirm that the concentration was 4 HAU/50 μL. Treated serum was serially diluted, mixed with 4 HAU antigen, and assessed for hemagglutination inhibition using 1% chicken RBCs.

### Urinalysis

Urine was collected using metabolic cages during a 4-h fasting period (± 1 h). Samples were analyzed promptly using dry chemistry methods on an N-600 urine analyzer. Parameters measured included urobilinogen (URO), bilirubin (BIL), ketones (KET), blood (BLD), protein (PRO), nitrite (NIT), leukocytes (LEU), glucose (GLU), specific gravity (SG), pH, vitamin C (VC), and microalbumin (MALB).

### Necropsy and histopathology

A complete gross necropsy and histopathological examination were performed on all rats in each study. Animals were anesthetized with 1% sodium pentobarbital (50 mg/kg, intraperitoneal) and exsanguinated via the abdominal aorta. External body surface, skin, mucous membranes, natural orifices, cranial cavity, thoracic cavity, abdominal cavity, and pelvic cavity were examined. All organs and tissues were collected according to the study protocol and relevant standard operating procedures. Eyes were fixed in Davidson’s fixative; all other tissues were fixed in 10% neutral buffered formalin. Tissues were trimmed, dehydrated, embedded in paraffin, sectioned at 3 μm, and stained with hematoxylin and eosin (H&E). All slides were examined microscopically by a board-certified pathologist. Findings were graded on a semi-quantitative scale: 0 = no lesion, 1 = minimal, 2 = mild, 3 = moderate, 4 = marked, 5 = severe; “P” indicated the presence of a lesion without a grade assignment. A peer reviewer confirmed and re-evaluated all histopathological observations.

### Statistical analysis

Data were analyzed using SPSS Statistics v23.0 (IBM Corp, Armonk, NY, USA). Continuous variables were expressed as the mean ± SD and compared using one-way ANOVA followed by Fisher’s Least Significant Difference (LSD) (homogeneous variance) or Games–Howell test (heterogeneous variance). Continuous variables with a non-normal distribution were analyzed using the Kruskal–Wallis test. P values< 0.05 were considered statistically significant.

## Data Availability

The original contributions presented in the study are included in the article/**Supplementary Material**. Further inquiries can be directed to the corresponding author/s.
