# Supplementary material for: Favorable safety and immunogenicity of a combined quadrivalent influenza and recombinant SARS-CoV-2 vaccine in Sprague-Dawley rats for both primary and booster immunization
Source: Front Immunol. 2026 Jul 17;17:1849279. doi: 10.3389/fimmu.2026.1849279 (PMC13424194; doi:10.3389/fimmu.2026.1849279)
Supplement: Supplementary file 1 [file Supplementaryfile1.docx]

**Supplementary table 1 The Organ weight and coefficient of male SD rats treated with Flu-CoV2 vaccine in Study 1.**

| **Parameter** | | **D31** | | | | | | | | | **D43** | | | | | | | | |
| --- | --- | --- | --- | --- | --- | --- | --- | --- | --- | --- | --- | --- | --- | --- | --- | --- | --- | --- | --- |
|  |  | **Control** | | | **Adjuvant** | | | **Flu-CoV2 vaccine** | | | **Control** | | | **Adjuvant** | | | **Flu-CoV2 vaccine** | | |
| Number of animals | | 10 | | | 10 | | | 10 | | | 5 | | | 5 | | | 5 | | |
| Body weight (g) | | 414 | ± | 27 | 421 | ± | 42 | 421 | ± | 26 | 473 | ± | 46 | 483 | ± | 61 | 471 | ± | 41 |
| **Brain** | Weight (g) | 2.201 | ± | 0.119 | 2.130 | ± | 0.108 | 2.168 | ± | 0.093 | 2.269 | ± | 0.088 | 2.287 | ± | 0.089 | 2.236 | ± | 0.033 |
|  | Organ coefficient (%) | 0.534 | ± | 0.040 | 0.509 | ± | 0.049 | 0.517 | ± | 0.038 | 0.482 | ± | 0.038 | 0.481 | ± | 0.073 | 0.478 | ± | 0.043 |
| **Heart** | Weight (g) | 1.482 | ± | 0.153 | 1.398 | ± | 0.109 | 1.543 | ± | 0.233 | 1.537 | ± | 0.185 | 1.620 | ± | 0.173 | 1.586 | ± | 0.199 |
|  | Organ coefficient (%) | 0.359 | ± | 0.038 | 0.333 | ± | 0.024 | 0.365 | ± | 0.036 | 0.325 | ± | 0.027 | 0.337 | ± | 0.021 | 0.336 | ± | 0.015 |
| **Liver** | Weight (g) | 11.057 | ± | 1.231 | 11.046 | ± | 1.505 | 12.016 | ± | 1.469 | 13.038 | ± | 1.957 | 13.263 | ± | 1.837 | 12.985 | ± | 2.398 |
|  | Organ coefficient (%) | 2.673 | ± | 0.232 | 2.616 | ± | 0.154 | 2.848 | ± | 0.204^△^ | 2.746 | ± | 0.164 | 2.745 | ± | 0.083 | 2.757 | ± | 0.461 |
| **Spleen** | Weight (g) | 0.807 | ± | 0.125 | 0.786 | ± | 0.160 | 0.827 | ± | 0.126 | 0.941 | ± | 0.167 | 0.984 | ± | 0.164 | 0.913 | ± | 0.156 |
|  | Organ coefficient (%) | 0.195 | ± | 0.029 | 0.185 | ± | 0.025 | 0.197 | ± | 0.028 | 0.199 | ± | 0.032 | 0.203 | ± | 0.014 | 0.193 | ± | 0.021 |
| **Kidney** | Weight (g) | 2.879 | ± | 0.131 | 2.919 | ± | 0.370 | 2.982 | ± | 0.341 | 3.176 | ± | 0.494 | 3.238 | ± | 0.333 | 3.053 | ± | 0.355 |
|  | Organ coefficient (%) | 0.699 | ± | 0.053 | 0.692 | ± | 0.036 | 0.707 | ± | 0.047 | 0.669 | ± | 0.053 | 0.673 | ± | 0.037 | 0.649 | ± | 0.048 |
| **Thymus** | Weight (g) | 0.433 | ± | 0.103 | 0.499 | ± | 0.101 | 0.422 | ± | 0.103 | 0.504 | ± | 0.167 | 0.454 | ± | 0.141 | 0.474 | ± | 0.095 |
|  | Organ coefficient (%) | 0.105 | ± | 0.022 | 0.118 | ± | 0.017 | 0.101 | ± | 0.027 | 0.108 | ± | 0.039 | 0.093 | ± | 0.023 | 0.101 | ± | 0.020 |
| **Adrenal gland** | Weight (g) | 0.064 | ± | 0.008 | 0.058 | ± | 0.014 | 0.065 | ± | 0.010 | 0.075 | ± | 0.005 | 0.066 | ± | 0.017 | 0.068 | ± | 0.017 |
|  | Organ coefficient (%) | 0.015 | ± | 0.002 | 0.014 | ± | 0.003 | 0.015 | ± | 0.002 | 0.016 | ± | 0.001 | 0.014 | ± | 0.003 | 0.014 | ± | 0.003 |
| **Testis** | Weight (g) | 3.373 | ± | 0.264 | 3.152 | ± | 0.273 | 3.391 | ± | 0.368 | 3.359 | ± | 0.280 | 3.492 | ± | 0.352 | 3.353 | ± | 0.441 |
|  | Organ coefficient (%) | 0.819 | ± | 0.086 | 0.752 | ± | 0.066 | 0.808 | ± | 0.095 | 0.714 | ± | 0.076 | 0.727 | ± | 0.058 | 0.711 | ± | 0.048 |
| **Epididymis** | Weight (g) | 1.199 | ± | 0.096 | 1.148 | ± | 0.093 | 1.193 | ± | 0.118 | 1.377 | ± | 0.165 | 1.334 | ± | 0.146 | 1.335 | ± | 0.243 |
|  | Organ coefficient (%) | 0.291 | ± | 0.028 | 0.275 | ± | 0.029 | 0.284 | ± | 0.025 | 0.292 | ± | 0.030 | 0.278 | ± | 0.022 | 0.282 | ± | 0.032 |

Note: The data were expressed as mean ± SD. ^△^P<0.05 vs adjuvant group. Organ coefficient (%) = g organ weight/g body weight×100%.

**Supplementary table 2 The Organ weight and coefficient of female SD rats treated with Flu-CoV2 vaccine in Study 1.**

| **Parameter** | | **D31** | | | | | | | | | **D43** | | | | | | | | | |
| --- | --- | --- | --- | --- | --- | --- | --- | --- | --- | --- | --- | --- | --- | --- | --- | --- | --- | --- | --- | --- |
|  |  | **Control** | | | **Adjuvant** | | | **Flu-CoV2 vaccine** | | | **Control** | | | **Adjuvant** | | | **Flu-CoV2 vaccine** | | | |
| Number of animals | | 10 | | | 10 | | | 10 | | | 5 | | | 5 | | | 5 | | | |
| Body weight (g) | | 246 | ± | 13 | 255 | ± | 17 | 250 | ± | 16 | 257 | ± | 18 | 268 | ± | 18 | 271 | ± | 37 |  |
| **Brain** | Weight (g) | 2.009 | ± | 0.084 | 2.048 | ± | 0.080 | 2.055 | ± | 0.083 | 2.014 | ± | 0.086 | 2.078 | ± | 0.142 | 2.047 | ± | 0.067 |  |
|  | Organ coefficient (%) | 0.817 | ± | 0.049 | 0.806 | ± | 0.055 | 0.827 | ± | 0.071 | 0.787 | ± | 0.056 | 0.775 | ± | 0.041 | 0.766 | ± | 0.096 |  |
| **Heart** | Weight (g) | 0.926 | ± | 0.072 | 0.994 | ± | 0.121 | 0.978 | ± | 0.047 | 0.954 | ± | 0.062 | 0.924 | ± | 0.097 | 0.894 | ± | 0.049 |  |
|  | Organ coefficient (%) | 0.376 | ± | 0.026 | 0.390 | ± | 0.044 | 0.393 | ± | 0.024 | 0.372 | ± | 0.012 | 0.344 | ± | 0.026 | 0.334 | ± | 0.037 |  |
| **Liver** | Weight (g) | 7.420 | ± | 0.966 | 7.537 | ± | 0.880 | 7.458 | ± | 0.379 | 7.519 | ± | 1.242 | 7.844 | ± | 0.518 | 7.430 | ± | 0.943 |  |
|  | Organ coefficient (%) | 3.003 | ± | 0.263 | 2.955 | ± | 0.220 | 2.993 | ± | 0.120 | 2.914 | ± | 0.296 | 2.926 | ± | 0.159 | 2.764 | ± | 0.338 |  |
| **Spleen** | Weight (g) | 0.601 | ± | 0.082 | 0.628 | ± | 0.067 | 0.639 | ± | 0.074 | 0.595 | ± | 0.097 | 0.581 | ± | 0.064 | 0.582 | ± | 0.111 |  |
|  | Organ coefficient (%) | 0.244 | ± | 0.032 | 0.248 | ± | 0.034 | 0.256 | ± | 0.026 | 0.231 | ± | 0.028 | 0.216 | ± | 0.016 | 0.216 | ± | 0.033 |  |
| **Kidney** | Weight (g) | 1.821 | ± | 0.143 | 1.890 | ± | 0.214 | 1.846 | ± | 0.100 | 1.861 | ± | 0.181 | 1.830 | ± | 0.130 | 1.829 | ± | 0.173 |  |
|  | Organ coefficient (%) | 0.739 | ± | 0.049 | 0.741 | ± | 0.056 | 0.741 | ± | 0.038 | 0.725 | ± | 0.048 | 0.682 | ± | 0.016 | 0.685 | ± | 0.107 |  |
| **Thymus** | Weight (g) | 0.343 | ± | 0.093 | 0.369 | ± | 0.043 | 0.361 | ± | 0.061 | 0.332 | ± | 0.055 | 0.326 | ± | 0.080 | 0.289 | ± | 0.068 |  |
|  | Organ coefficient (%) | 0.139 | ± | 0.038 | 0.145 | ± | 0.021 | 0.144 | ± | 0.020 | 0.130 | ± | 0.024 | 0.121 | ± | 0.024 | 0.109 | ± | 0.031 |  |
| **Adrenal gland** | Weight (g) | 0.065 | ± | 0.007 | 0.067 | ± | 0.009 | 0.066 | ± | 0.009 | 0.068 | ± | 0.008 | 0.063 | ± | 0.005 | 0.072 | ± | 0.009 |  |
|  | Organ coefficient (%) | 0.026 | ± | 0.002 | 0.026 | ± | 0.004 | 0.026 | ± | 0.003 | 0.026 | ± | 0.003 | 0.024 | ± | 0.002 | 0.027 | ± | 0.004 |  |
| **Uterus** | Weight (g) | 0.719 | ± | 0.197 | 0.905 | ± | 0.292 | 0.705 | ± | 0.240 | 0.736 | ± | 0.121 | 0.856 | ± | 0.380 | 0.715 | ± | 0.176 |  |
|  | Organ coefficient (%) | 0.293 | ± | 0.085 | 0.360 | ± | 0.129 | 0.285 | ± | 0.106 | 0.287 | ± | 0.041 | 0.324 | ± | 0.153 | 0.265 | ± | 0.058 |  |
| **Ovary** | Weight (g) | 0.081 | ± | 0.015 | 0.094 | ± | 0.020 | 0.093 | ± | 0.020 | 0.077 | ± | 0.020 | 0.089 | ± | 0.027 | 0.088 | ± | 0.016 |  |
|  | Organ coefficient (%) | 0.033 | ± | 0.006 | 0.037 | ± | 0.007 | 0.037 | ± | 0.008 | 0.030 | ± | 0.007 | 0.033 | ± | 0.009 | 0.033 | ± | 0.005 |  |

Note: The data were expressed as mean ± SD. Organ coefficient (%) = g organ weight/g body weight×100%.

**Supplementary table 3 The Organ weight and coefficient of male SD rats treated with Flu-CoV2 Vaccine in Study 2.**

| **Parameter** | | **D74** | | | | | | | | | **D85** | | | | | | | | |
| --- | --- | --- | --- | --- | --- | --- | --- | --- | --- | --- | --- | --- | --- | --- | --- | --- | --- | --- | --- |
|  |  | **Control** | | | **Flu vaccine+Saline** | | | **Flu+** **Flu-CoV2 vaccine** | | | **Control** | | | **Flu vaccine+Saline** | | | **Flu+** **Flu-CoV2 vaccine** | | |
| Number of animals | | 10 | | | 10 | | | 10 | | | 5 | | | 5 | | | 5 | | |
| Body weight (g) | | 476 | ± | 31 | 517 | ± | 62 | 484 | ± | 56 | 515 | ± | 38 | 540 | ± | 59 | 514 | ± | 34 |
| **Brain** | Weight (g) | 1.614 | ± | 0.194 | 1.696 | ± | 0.235 | 1.576 | ± | 1.087 | 1.466 | ± | 0.318 | 1.627 | ± | 0.125 | 1.526 | ± | 0.048 |
|  | Organ coefficient (%) | 0.338 | ± | 0.028 | 0.328 | ± | 0.027 | 0.326 | ± | 0.355 | 0.283 | ± | 0.046 | 0.304 | ± | 0.037 | 0.298 | ± | 0.016 |
| **Heart** | Weight (g) | 11.799 | ± | 0.913 | 13.413 | ± | 3.052 | 12.390 | ± | 8.496 | 13.940 | ± | 1.845 | 14.738 | ± | 1.941 | 13.722 | ± | 1.387 |
|  | Organ coefficient (%) | 2.479 | ± | 0.137 | 2.567 | ± | 0.270 | 2.562 | ± | 2.765 | 2.701 | ± | 0.198 | 2.733 | ± | 0.215 | 2.669 | ± | 0.206 |
| **Liver** | Weight (g) | 0.887 | ± | 0.166 | 0.935 | ± | 0.109 | 0.973 | ± | 0.667 | 0.979 | ± | 0.278 | 0.847 | ± | 0.058 | 0.824 | ± | 0.106 |
|  | Organ coefficient (%) | 0.185 | ± | 0.027 | 0.182 | ± | 0.027 | 0.201 | ± | 0.217 | 0.190 | ± | 0.049 | 0.158 | ± | 0.020 | 0.161 | ± | 0.028 |
| **Spleen** | Weight (g) | 3.056 | ± | 0.295 | 3.408 | ± | 0.436 | 3.194 | ± | 2.067 | 3.237 | ± | 0.478 | 3.342 | ± | 0.444 | 3.325 | ± | 0.289 |
|  | Organ coefficient (%) | 0.641 | ± | 0.039 | 0.659 | ± | 0.042 | 0.661 | ± | 0.674 | 0.627 | ± | 0.061 | 0.619 | ± | 0.039 | 0.648 | ± | 0.048 |
| **Kidney** | Weight (g) | 2.167 | ± | 0.169 | 2.290 | ± | 0.104 | 2.263 | ± | 2.188 | 2.230 | ± | 0.146 | 2.221 | ± | 0.129 | 2.231 | ± | 0.119 |
|  | Organ coefficient (%) | 0.456 | ± | 0.032 | 0.447 | ± | 0.044 | 0.473 | ± | 0.718 | 0.434 | ± | 0.028 | 0.414 | ± | 0.035 | 0.435 | ± | 0.029 |
| **Thymus** | Weight (g) | 0.345 | ± | 0.110 | 0.374 | ± | 0.116 | 0.295 | ± | 0.312 | 0.407 | ± | 0.113 | 0.310 | ± | 0.055 | 0.300 | ± | 0.060 |
|  | Organ coefficient (%) | 0.072 | ± | 0.020 | 0.071 | ± | 0.013 | 0.061 | ± | 0.102 | 0.080 | ± | 0.024 | 0.057 | ± | 0.007 | 0.058 | ± | 0.011 |
| **Adrenal gland** | Weight (g) | 0.061 | ± | 0.012 | 0.067 | ± | 0.013 | 0.067 | ± | 0.075 | 0.055 | ± | 0.004 | 0.054 | ± | 0.010 | 0.061 | ± | 0.005 |
|  | Organ coefficient (%) | 0.013 | ± | 0.002 | 0.013 | ± | 0.002 | 0.014 | ± | 0.024 | 0.011 | ± | 0.001 | 0.010 | ± | 0.001 | 0.012 | ± | 0.001 |
| **Testis** | Weight (g) | 3.258 | ± | 0.216 | 3.582 | ± | 0.351 | 3.265 | ± | 0.876 | 3.127 | ± | 0.906 | 3.219 | ± | 0.278 | 3.398 | ± | 0.285 |
|  | Organ coefficient (%) | 0.686 | ± | 0.049 | 0.696 | ± | 0.059 | 0.679 | ± | 0.284 | 0.601 | ± | 0.153 | 0.605 | ± | 0.099 | 0.661 | ± | 0.028 |
| **Epididymis** | Weight (g) | 1.346 | ± | 0.081 | 1.440 | ± | 0.111 | 1.431 | ± | 0.088 | 1.411 | ± | 0.285 | 1.393 | ± | 0.079 | 1.477 | ± | 0.128 |
|  | Organ coefficient (%) | 0.283 | ± | 0.018 | 0.281 | ± | 0.031 | 0.298 | ± | 0.028 | 0.272 | ± | 0.040 | 0.261 | ± | 0.036 | 0.287 | ± | 0.009 |

Note: The data were expressed as mean ± SD. Organ coefficient (%) = g organ weight/g body weight×100%.

**Supplementary table 4 The Organ weight and coefficient of female SD rats treated with Flu-CoV2 Vaccine in Study 2.**

| **Parameter** | | **D74** | | | | | | | | | **D85** | | | | | | | | | |
| --- | --- | --- | --- | --- | --- | --- | --- | --- | --- | --- | --- | --- | --- | --- | --- | --- | --- | --- | --- | --- |
|  |  | **Control** | | | **Flu vaccine+Saline** | | | **Flu+** **Flu-CoV2 vaccine** | | | **Control** | | | **Flu vaccine+Saline** | | | **Flu+** **Flu-CoV2 vaccine** | | | |
| Number of animals | | 10 | | | 10 | | | 10 | | | 5 | | | 5 | | | 5 | | | |
| Body weight (g) | | 307 | ± | 26 | 303 | ± | 32 | 304 | ± | 22 | 325 | ± | 30 | 314 | ± | 37 | 324 | ± | 29 |  |
| **Brain** | Weight (g) | 1.087 | ± | 0.117 | 1.108 | ± | 0.193 | 1.122 | ± | 0.127 | 1.103 | ± | 0.079 | 1.030 | ± | 0.088 | 1.034 | ± | 0.064 |  |
|  | Organ coefficient (%) | 0.355 | ± | 0.029 | 0.365 | ± | 0.046 | 0.369 | ± | 0.032 | 0.342 | ± | 0.037 | 0.330 | ± | 0.028 | 0.320 | ± | 0.015 |  |
| **Heart** | Weight (g) | 8.496 | ± | 1.098 | 8.184 | ± | 1.162 | 8.231 | ± | 0.738 | 9.151 | ± | 0.598 | 9.240 | ± | 1.353 | 9.457 | ± | 1.326 |  |
|  | Organ coefficient (%) | 2.765 | ± | 0.246 | 2.694 | ± | 0.232 | 2.710 | ± | 0.154 | 2.837 | ± | 0.297 | 2.943 | ± | 0.218 | 2.914 | ± | 0.313 |  |
| **Liver** | Weight (g) | 0.667 | ± | 0.079 | 0.683 | ± | 0.128 | 0.670 | ± | 0.127 | 0.632 | ± | 0.077 | 0.655 | ± | 0.082 | 0.630 | ± | 0.080 |  |
|  | Organ coefficient (%) | 0.217 | ± | 0.016 | 0.224 | ± | 0.032 | 0.220 | ± | 0.035 | 0.196 | ± | 0.025 | 0.210 | ± | 0.027 | 0.196 | ± | 0.038 |  |
| **Spleen** | Weight (g) | 2.067 | ± | 0.193 | 2.131 | ± | 0.326 | 2.119 | ± | 0.158 | 2.048 | ± | 0.130 | 2.080 | ± | 0.133 | 2.022 | ± | 0.160 |  |
|  | Organ coefficient (%) | 0.674 | ± | 0.042 | 0.703 | ± | 0.084 | 0.700 | ± | 0.062 | 0.634 | ± | 0.057 | 0.668 | ± | 0.066 | 0.626 | ± | 0.057 |  |
| **Kidney** | Weight (g) | 2.188 | ± | 0.103 | 2.169 | ± | 0.079 | 2.146 | ± | 0.067 | 2.157 | ± | 0.042 | 2.051 | ± | 0.066 | 2.075 | ± | 0.090 |  |
|  | Organ coefficient (%) | 0.718 | ± | 0.072 | 0.721 | ± | 0.066 | 0.709 | ± | 0.049 | 0.669 | ± | 0.059 | 0.661 | ± | 0.074 | 0.642 | ± | 0.034 |  |
| **Thymus** | Weight (g) | 0.312 | ± | 0.055 | 0.291 | ± | 0.078 | 0.314 | ± | 0.048 | 0.302 | ± | 0.028 | 0.323 | ± | 0.095 | 0.327 | ± | 0.054 |  |
|  | Organ coefficient (%) | 0.102 | ± | 0.017 | 0.096 | ± | 0.024 | 0.104 | ± | 0.019 | 0.093 | ± | 0.009 | 0.102 | ± | 0.022 | 0.102 | ± | 0.022 |  |
| **Adrenal gland** | Weight (g) | 0.075 | ± | 0.012 | 0.096 | ± | 0.059 | 0.075 | ± | 0.011 | 0.064 | ± | 0.012 | 0.073 | ± | 0.020 | 0.063 | ± | 0.012 |  |
|  | Organ coefficient (%) | 0.024 | ± | 0.004 | 0.033 | ± | 0.026 | 0.025 | ± | 0.003 | 0.020 | ± | 0.005 | 0.024 | ± | 0.008 | 0.020 | ± | 0.005 |  |
| **Uterus** | Weight (g) | 0.876 | ± | 0.239 | 1.154 | ± | 0.432 | 0.866 | ± | 0.086 | 0.775 | ± | 0.169 | 0.785 | ± | 0.126 | 0.705 | ± | 0.112 |  |
|  | Organ coefficient (%) | 0.284 | ± | 0.069 | 0.377 | ± | 0.125 | 0.286 | ± | 0.034 | 0.241 | ± | 0.056 | 0.250 | ± | 0.027 | 0.219 | ± | 0.040 |  |
| **Ovary** | Weight (g) | 0.088 | ± | 0.013 | 0.099 | ± | 0.022 | 0.101 | ± | 0.027 | 0.086 | ± | 0.021 | 0.101 | ± | 0.025 | 0.114 | ± | 0.025 |  |
|  | Organ coefficient (%) | 0.028 | ± | 0.003 | 0.033 | ± | 0.006 | 0.033 | ± | 0.008 | 0.026 | ± | 0.004 | 0.033 | ± | 0.009 | 0.035 | ± | 0.007 |  |

Note: The data were expressed as mean ± SD. Organ coefficient (%) = g organ weight/g body weight×100%.

**Supplementary table 5 The Organ weight and coefficient of male SD rats treated with Flu-CoV2 vaccine in Study 3.**

| **Parameter** | | **D101** | | | | | | | | | | | | | **D113** | | | | | | | | | | | | | | |
| --- | --- | --- | --- | --- | --- | --- | --- | --- | --- | --- | --- | --- | --- | --- | --- | --- | --- | --- | --- | --- | --- | --- | --- | --- | --- | --- | --- | --- | --- |
|  |  | **Control** | | | **CoV2 vaccine+Saline** | | | **CoV2+Flu vaccine** | | | **CoV2+Flu-CoV2 vaccine** | | | | **Control** | | | | **CoV2 vaccine+Saline** | | | | **CoV2+Flu vaccine** | | | | **CoV2+Flu-CoV2 vaccine** | | |
| Number of animals | | 10 | | | 10 | | | 10 | | | 10 | | | | 5 | | | | 5 | | | | 5 | | | | 5 | | |
| Body weight (g) | | 539 | ± | 25 | 573 | ± | 30 | 564 | ± | 50 | 568 | ± | 61 | 593 | | ± | 69 | 599 | | ± | 36 | 607 | | ± | 71 | 620 | | ± | 63 |
| **Brain** | Weight (g) | 1.615 | ± | 0.126 | 1.703 | ± | 0.127 | 1.768 | ± | 0.262 | 1.713 | ± | 0.205 | 1.676 | | ± | 0.277 | 1.853 | | ± | 0.205 | 1.734 | | ± | 0.254 | 1.800 | | ± | 0.322 |
|  | Organ coefficient (%) | 0.300 | ± | 0.024 | 0.298 | ± | 0.022 | 0.313 | ± | 0.033 | 0.301 | ± | 0.012 | 0.285 | | ± | 0.053 | 0.309 | | ± | 0.030 | 0.287 | | ± | 0.043 | 0.289 | | ± | 0.022 |
| **Heart** | Weight (g) | 12.527 | ± | 0.765 | 13.322 | ± | 1.578 | 13.699 | ± | 1.395 | 13.746 | ± | 1.798 | 15.635 | | ± | 2.371 | 15.991 | | ± | 1.231 | 17.065 | | ± | 2.586 | 18.027 | | ± | 4.273 |
|  | Organ coefficient (%) | 2.327 | ± | 0.133 | 2.325 | ± | 0.233 | 2.431 | ± | 0.174 | 2.427 | ± | 0.296 | 2.633 | | ± | 0.248 | 2.668 | | ± | 0.126 | 2.807 | | ± | 0.158 | 2.885 | | ± | 0.456 |
| **Liver** | Weight (g) | 0.897 | ± | 0.104 | 0.978 | ± | 0.119 | 0.943 | ± | 0.094 | 1.019 | ± | 0.185 | 0.931 | | ± | 0.147 | 0.991 | | ± | 0.168 | 0.963 | | ± | 0.095 | 1.132 | | ± | 0.084 |
|  | Organ coefficient (%) | 0.166 | ± | 0.017 | 0.171 | ± | 0.018 | 0.167 | ± | 0.012 | 0.178 | ± | 0.017 | 0.157 | | ± | 0.019 | 0.166 | | ± | 0.027 | 0.159 | | ± | 0.011 | 0.183 | | ± | 0.010 |
| **Spleen** | Weight (g) | 3.274 | ± | 0.472 | 3.328 | ± | 0.223 | 3.422 | ± | 0.465 | 3.229 | ± | 0.372 | 3.501 | | ± | 0.318 | 3.634 | | ± | 0.169 | 3.695 | | ± | 0.401 | 3.819 | | ± | 0.544 |
|  | Organ coefficient (%) | 0.607 | ± | 0.081 | 0.582 | ± | 0.042 | 0.606 | ± | 0.059 | 0.570 | ± | 0.049 | 0.592 | | ± | 0.045 | 0.608 | | ± | 0.043 | 0.611 | | ± | 0.050 | 0.615 | | ± | 0.036 |
| **Kidney** | Weight (g) | 2.345 | ± | 0.099 | 2.331 | ± | 0.055 | 2.318 | ± | 0.094 | 2.309 | ± | 0.122 | 2.255 | | ± | 0.075 | 2.393 | | ± | 0.118 | 3.472 | | ± | 0.864 | 2.462 | | ± | 0.164 |
|  | Organ coefficient (%) | 0.436 | ± | 0.025 | 0.408 | ± | 0.028 | 0.413 | ± | 0.034 | 0.409 | ± | 0.029 | 0.383 | | ± | 0.033 | 0.400 | | ± | 0.022 | 0.570 | | ± | 0.125 | 0.399 | | ± | 0.026 |
| **Thymus** | Weight (g) | 0.297 | ± | 0.082 | 0.312 | ± | 0.110 | 0.313 | ± | 0.089 | 0.335 | ± | 0.069 | 0.301 | | ± | 0.086 | 0.346 | | ± | 0.050 | 0.281 | | ± | 0.125 | 0.299 | | ± | 0.047 |
|  | Organ coefficient (%) | 0.055 | ± | 0.014 | 0.054 | ± | 0.018 | 0.056 | ± | 0.016 | 0.059 | ± | 0.013 | 0.051 | | ± | 0.012 | 0.058 | | ± | 0.011 | 0.045 | | ± | 0.014 | 0.048 | | ± | 0.006 |
| **Adrenal gland** | Weight (g) | 0.054 | ± | 0.006 | 0.066 | ± | 0.009* | 0.067 | ± | 0.012** | 0.058 | ± | 0.010 | 0.054 | | ± | 0.007 | 0.060 | | ± | 0.012 | 0.062 | | ± | 0.004 | 0.061 | | ± | 0.004 |
|  | Organ coefficient (%) | 0.010 | ± | 0.001 | 0.011 | ± | 0.001 | 0.012 | ± | 0.002* | 0.010 | ± | 0.001^■^ | 0.009 | | ± | 0.001 | 0.010 | | ± | 0.003 | 0.010 | | ± | 0.001 | 0.010 | | ± | 0.001 |
| **Testis** | Weight (g) | 3.554 | ± | 0.440 | 3.613 | ± | 0.285 | 3.443 | ± | 0.326 | 3.579 | ± | 0.344 | 3.394 | | ± | 0.428 | 3.731 | | ± | 0.146 | 3.998 | | ± | 0.697 | 3.639 | | ± | 0.514 |
|  | Organ coefficient (%) | 0.660 | ± | 0.075 | 0.639 | ± | 0.052 | 0.611 | ± | 0.042 | 0.633 | ± | 0.057 | 0.577 | | ± | 0.090 | 0.624 | | ± | 0.040 | 0.662 | | ± | 0.110 | 0.596 | | ± | 0.123 |
| **Epididymis** | Weight (g) | 1.414 | ± | 0.136 | 1.844 | ± | 1.234 | 1.506 | ± | 0.200 | 1.517 | ± | 0.138 | 1.462 | | ± | 0.127 | 1.528 | | ± | 0.076 | 1.743 | | ± | 0.337 | 1.562 | | ± | 0.208 |
|  | Organ coefficient (%) | 0.263 | ± | 0.025 | 0.322 | ± | 0.198 | 0.267 | ± | 0.027 | 0.269 | ± | 0.028 | 0.248 | | ± | 0.027 | 0.256 | | ± | 0.026 | 0.288 | | ± | 0.051 | 0.256 | | ± | 0.053 |

Note: The data were expressed as mean ± SD. *P<0.05, **P<0.01 vs control group; ^■^P<0.05 vs CoV2+Flu vaccine group. Organ coefficient (%) = g organ weight/g body weight×100%.

**Supplementary table 6 The Organ weight and coefficient of female SD rats treated with Flu-CoV2 vaccine in Study 3.**

| **Parameter** | | **D101** | | | | | | | | | | | | **D113** | | | | | | | | | | | |
| --- | --- | --- | --- | --- | --- | --- | --- | --- | --- | --- | --- | --- | --- | --- | --- | --- | --- | --- | --- | --- | --- | --- | --- | --- | --- |
|  |  | **Control** | | | **CoV2 vaccine+Saline** | | | **CoV2+Flu vaccine** | | | **CoV2+Flu-CoV2 vaccine** | | | **Control** | | | **CoV2 vaccine+Saline** | | | **CoV2+Flu vaccine** | | | **CoV2+Flu-CoV2 vaccine** | | |
| Number of animals | | 10 | | | 10 | | | 10 | | | 10 | | | 5 | | | 5 | | | 5 | | | 5 | | |
| Body weight (g) | | 304 | ± | 38 | 288 | ± | 30 | 315 | ± | 31 | 305 | ± | 39 | 325 | ± | 49 | 307 | ± | 44 | 322 | ± | 44 | 320 | ± | 29 |
| **Brain** | Weight (g) | 1.038 | ± | 0.111 | 1.002 | ± | 0.133 | 1.076 | ± | 0.109 | 1.048 | ± | 0.140 | 0.993 | ± | 0.176 | 1.095 | ± | 0.231 | 1.076 | ± | 0.111 | 1.106 | ± | 0.110 |
|  | Organ coefficient (%) | 0.342 | ± | 0.017 | 0.348 | ± | 0.026 | 0.343 | ± | 0.029 | 0.344 | ± | 0.022 | 0.306 | ± | 0.035 | 0.356 | ± | 0.041 | 0.337 | ± | 0.030 | 0.348 | ± | 0.050 |
| **Heart** | Weight (g) | 8.047 | ± | 1.137 | 7.946 | ± | 1.181 | 8.257 | ± | 0.943 | 8.163 | ± | 0.960 | 9.437 | ± | 1.705 | 9.131 | ± | 1.309 | 9.091 | ± | 1.429 | 9.898 | ± | 1.242 |
|  | Organ coefficient (%) | 2.648 | ± | 0.246 | 2.755 | ± | 0.217 | 2.625 | ± | 0.229 | 2.686 | ± | 0.235 | 2.897 | ± | 0.276 | 2.979 | ± | 0.218 | 2.823 | ± | 0.175 | 3.084 | ± | 0.121 |
| **Liver** | Weight (g) | 0.585 | ± | 0.063 | 0.541 | ± | 0.087 | 0.630 | ± | 0.083 | 0.609 | ± | 0.112 | 0.538 | ± | 0.123 | 0.554 | ± | 0.094 | 0.583 | ± | 0.067 | 0.617 | ± | 0.068 |
|  | Organ coefficient (%) | 0.193 | ± | 0.022 | 0.189 | ± | 0.029 | 0.200 | ± | 0.022 | 0.199 | ± | 0.027 | 0.165 | ± | 0.025 | 0.180 | ± | 0.009 | 0.183 | ± | 0.019 | 0.193 | ± | 0.015 |
| **Spleen** | Weight (g) | 1.967 | ± | 0.273 | 1.982 | ± | 0.174 | 2.043 | ± | 0.136 | 2.060 | ± | 0.254 | 2.007 | ± | 0.355 | 2.167 | ± | 0.472 | 2.164 | ± | 0.214 | 2.109 | ± | 0.163 |
|  | Organ coefficient (%) | 0.647 | ± | 0.060 | 0.692 | ± | 0.055 | 0.652 | ± | 0.051 | 0.679 | ± | 0.071 | 0.616 | ± | 0.038 | 0.703 | ± | 0.082 | 0.678 | ± | 0.066 | 0.660 | ± | 0.038 |
| **Kidney** | Weight (g) | 2.145 | ± | 0.074 | 2.069 | ± | 0.084 | 2.121 | ± | 0.081 | 2.129 | ± | 0.050 | 2.101 | ± | 0.047 | 2.060 | ± | 0.151 | 2.144 | ± | 0.209 | 2.096 | ± | 0.070 |
|  | Organ coefficient (%) | 0.713 | ± | 0.083 | 0.726 | ± | 0.079 | 0.679 | ± | 0.065 | 0.706 | ± | 0.076 | 0.656 | ± | 0.087 | 0.678 | ± | 0.076 | 0.672 | ± | 0.069 | 0.658 | ± | 0.047 |
| **Thymus** | Weight (g) | 0.248 | ± | 0.056 | 0.250 | ± | 0.052 | 0.268 | ± | 0.035 | 0.247 | ± | 0.062 | 0.260 | ± | 0.065 | 0.244 | ± | 0.048 | 0.229 | ± | 0.050 | 0.251 | ± | 0.072 |
|  | Organ coefficient (%) | 0.082 | ± | 0.017 | 0.087 | ± | 0.018 | 0.086 | ± | 0.017 | 0.080 | ± | 0.015 | 0.081 | ± | 0.023 | 0.080 | ± | 0.012 | 0.071 | ± | 0.008 | 0.078 | ± | 0.018 |
| **Adrenal gland** | Weight (g) | 0.070 | ± | 0.016 | 0.075 | ± | 0.015 | 0.065 | ± | 0.011 | 0.075 | ± | 0.014 | 0.065 | ± | 0.010 | 0.076 | ± | 0.015 | 0.071 | ± | 0.004 | 0.071 | ± | 0.007 |
|  | Organ coefficient (%) | 0.023 | ± | 0.005 | 0.026 | ± | 0.005 | 0.021 | ± | 0.003^▲▲^ | 0.025 | ± | 0.004^■^ | 0.020 | ± | 0.004 | 0.025 | ± | 0.004 | 0.022 | ± | 0.004 | 0.022 | ± | 0.003 |
| **Uterus** | Weight (g) | 0.826 | ± | 0.340 | 0.856 | ± | 0.164 | 0.902 | ± | 0.259 | 0.957 | ± | 0.487 | 1.108 | ± | 0.900 | 0.929 | ± | 0.257 | 0.864 | ± | 0.186 | 1.116 | ± | 0.532 |
|  | Organ coefficient (%) | 0.277 | ± | 0.125 | 0.299 | ± | 0.059 | 0.293 | ± | 0.103 | 0.316 | ± | 0.172 | 0.319 | ± | 0.196 | 0.305 | ± | 0.088 | 0.270 | ± | 0.054 | 0.346 | ± | 0.156 |
| **Ovary** | Weight (g) | 0.091 | ± | 0.021 | 0.077 | ± | 0.016 | 0.087 | ± | 0.016 | 0.081 | ± | 0.019 | 0.089 | ± | 0.021 | 0.097 | ± | 0.035 | 0.097 | ± | 0.012 | 0.096 | ± | 0.029 |
|  | Organ coefficient (%) | 0.030 | ± | 0.008 | 0.027 | ± | 0.005 | 0.028 | ± | 0.005 | 0.027 | ± | 0.006 | 0.028 | ± | 0.006 | 0.032 | ± | 0.011 | 0.031 | ± | 0.006 | 0.030 | ± | 0.007 |

Note: The data were expressed as mean ± SD. ^▲▲^P<0.01 vs COVID-19 vaccine group; ^■^P<0.05 vs CoV2+Flu vaccine group. Organ coefficient (%) = g organ weight/g body weight×100%.

**Supplementary table 7 The urinalysis of male SD rats treated with Flu-CoV2 Vaccine in Study 1.**

| **Parameter** | | **D0** | | | **D31** | | | **D43** | | |
| --- | --- | --- | --- | --- | --- | --- | --- | --- | --- | --- |
|  |  | **Control** | **Adjuvant** | **Flu-CoV2 vaccine** | **Control** | **Adjuvant** | **Flu-CoV2 vaccine** | **Control** | **Adjuvant** | **Flu-CoV2 vaccine** |
| Number of animals | | 15 | 15 | 15 | 10 | 10 | 10 | 5 | 5 | 5 |
| Urobilinogen (μmol/L) | Normal | 15 | 15 | 15 | 10 | 10 | 10 | 5 | 5 | 5 |
| Bilirubin (μmol/L) | Neg | 15 | 15 | 15 | 10 | 10 | 10 | 5 | 5 | 5 |
| Ketone bodies (mmol/L) | Neg | 5 | 10 | 9 | / | / | / | 4 | 4 | 3 |
|  | 0.5 | 10 | 5 | 6 | 6 | 7 | 9 | 1 | 1 | 1 |
|  | 1.5 | / | / | / | 4 | 3 | 1 | / | / | 1 |
| Blood (Ery/μl) | Neg | 14 | 15 | 15 | 10 | 10 | 10 | 5 | 5 | 5 |
|  | ca.200 | 1 | / | / | / | / | / | / | / | / |
| Protein (g/L) | Neg | 14 | 15 | 15 | 3 | 5 | 7 | 5 | 4 | 5 |
|  | Trace | 1 | / | / | 6 | 3 | 3 | / | 1 | / |
|  | 0.3 | / | / | / | 1 | 2 | / | / | / | / |
| Nitrite | Neg | 15 | 15 | 15 | 10 | 10 | 10 | 5 | 5 | 5 |
| Leukocytes (Leuko/μL) | Neg | 7 | 10 | 8 | 2 | / | 2 | 4 | 4 | 3 |
|  | ca.15 | 7 | 5 | 6 | 2 | 6 | 6 | 1 | 1 | 2 |
|  | ca.70 | / | / | 1 | 6 | 4 | 2 | / | / | / |
|  | ca.125 | 1 | / | / | / | / | / | / | / | / |
| Glucose (mmol/L) | Neg | 15 | 15 | 15 | 10 | 10 | 10 | 5 | 5 | 5 |
| Microalbumin (g/L) | Neg | / | 3 | 3 | / | / | / | 3 | 2 | / |
|  | 0.15 | 14 | 12 | 12 | 3 | 5 | 7 | 2 | 2 | 5 |
|  | >0.15 | 1 | / | / | 7 | 5 | 3 | / | 1 | / |
| Specific gravity | <=1.005 | 10 | 14 | 10 | 4 | 1 | 5 | 4 | 4 | 4 |
|  | 1.010 | 4 | 1 | 5 | 1 | 6 | 3 | 1 | 1 | 1 |
|  | 1.015 | 1 | / | / | 4 | 2 | 2 | / | / | / |
|  | 1.020 | / | / | / | 1 | / | / | / | / | / |
|  | 1.250 | / | / | / | / | 1 | / | / | / | / |
| pH | 6.0 | / | / | / | / | / | / | / | / | 1 |
|  | 6.5 | / | / | / | 1 | 1 | / | / | 1 | / |
|  | 7.0 | / | 2 | 1 | 1 | 2 | / | / | / | / |
|  | 7.5 | 9 | 8 | 11 | 2 | 3 | 8 | 4 | 2 | 3 |
|  | 8.0 | 4 | 5 | 3 | 6 | 2 | 2 | 1 | 2 | 1 |
|  | 8.5 | 2 | / | / | / | 2 | / | / | / | / |
| Vitamin C (mmol/L) | 0.0 | 15 | 15 | 15 | 10 | 9 | 6 | 5 | 5 | 4 |
|  | 0.6 | / | / | / | / | 1 | 4 | 0 | 0 | 1 |

Note: “Normal” and “Neg” indicate no abnormality in the indicator.

**Supplementary table 8 The urinalysis of female SD rats treated with Flu-CoV2 vaccine in Study 1.**

| **Parameter** | | **D0** | | | **D31** | | | **D43** | | |
| --- | --- | --- | --- | --- | --- | --- | --- | --- | --- | --- |
|  |  | **Control** | **Adjuvant** | **Flu-CoV2 vaccine** | **Control** | **Adjuvant** | **Flu-CoV2 vaccine** | **Control** | **Adjuvant** | **Flu-CoV2 vaccine** |
| Number of animals | | 15 | 15 | 15 | 10 | 10 | 10 | 5 | 5 | 5 |
| Urobilinogen (μmol/L) | Normal | 15 | 15 | 15 | 10 | 10 | 10 | 5 | 5 | 5 |
| Bilirubin (μmol/L) | Neg | 15 | 15 | 15 | 10 | 10 | 10 | 5 | 5 | 5 |
| Ketone bodies (mmol/L) | Neg | 15 | 11 | 9* | 10 | 10 | 10 | 1 | 3 | 1 |
|  | 0.5 | / | 4 | 6 | / | / | / | 4 | 2 | 4 |
| Blood (Ery/μl) | Neg | 15 | 15 | 15 | 10 | 10 | 10 | 5 | 5 | 5 |
| Protein (g/L) | Neg | 15 | 15 | 14 | 10 | 10 | 10 | 4 | 5 | 5 |
|  | Trace | / | / | 1 | / | / | / | 1 | / | / |
| Nitrite | Neg | 15 | 15 | 15 | 10 | 10 | 10 | 5 | 4 | 5 |
|  | Pos | / | / | / | / | / | / | / | 1 | / |
| Leukocytes (Leuko/μL) | Neg | 14 | 9 | 9 | 9 | 10 | 9 | / | 3 | 3 |
|  | 15 | 1 | 5 | 6 | 1 | / | / | 3 | 2 | 2 |
|  | 70 | / | 1 | / | / | / | 1 | 2 | / | / |
| Glucose (mmol/L) | Neg | 15 | 15 | 15 | 10 | 10 | 10 | 5 | 5 | 5 |
| Microalbumin (g/L) | Neg | 9 | 5 | 4 | 1 | 3 | / | / | 1 | / |
|  | 0.15 | 6 | 10 | 10 | 9 | 7 | 10 | 4 | 4 | 5 |
|  | >0.15 | / | / | 1 | / | / | / | 1 | / | / |
| Specific gravity | <=1.005 | 15 | 12 | 13 | 9 | 10 | 9 | 4 | 5 | 5 |
|  | 1.010 | / | 3 | 2 | 1 | 0 | 1 | 1 | / | / |
| pH | 6.0 | / | / | / | / | / | / | 1 | / | / |
|  | 6.5 | / | / | 2 | 2 | 2 | 2 | / | / | / |
|  | 7.0 | / | / | 2 | 4 | 1 | 3 | / | / | 1 |
|  | 7.5 | 10 | 10 | 10 | 3 | 7 | 4 | 1 | 5 | 3 |
|  | 8.0 | 5 | 4 | 1 | 1 | / | 1 | 3 | / | 1 |
|  | 8.5 | / | 1 | / | / | / | / | / | / | / |
| Vitamin C (mmol/L) | 0.0 | 15 | 15 | 15 | 10 | 10 | 10 | 5 | 5 | 4 |
|  | 0.6 | / | / | / | / | / | / | / | / | 1 |

Note: “Normal” and “Neg” indicate no abnormality in the indicator. “POS” indicates a nitrite test result of any degree of uniform pink. “*” indicates a statistically significant difference at p <0.05 when compared to the control group

**Supplementary table 9 The urinalysis of male SD rats treated with Flu-CoV2 vaccine in Study 2.**

| **Parameter** | | **D0** | | | **D74** | | | **D85** | | |
| --- | --- | --- | --- | --- | --- | --- | --- | --- | --- | --- |
|  |  | **Control** | **Flu vaccine+Saline** | **Flu+** **Flu-CoV2 vaccine** | **Control** | **Flu vaccine** | **Flu+** **Flu-CoV2 vaccine** | **Control** | **Flu vaccine+Saline** | **Flu+** **Flu-CoV2 vaccine** |
| Number of animals | | 15 | 15 | 15 | 10 | 10 | 10 | 5 | 5 | 5 |
| Urobilinogen (μmol/L) | Normal | 15 | 15 | 15 | 10 | 10 | 10 | 5 | 5 | 5 |
| Bilirubin (μmol/L) | Neg | 15 | 15 | 15 | 10 | 10 | 10 | 5 | 5 | 5 |
| Ketone bodies (mmol/L) | Neg | 13 | 13 | 10 | 2 | 5 | 2 | 2 | 2 | 3 |
|  | 0.5 | 2 | 2 | 5 | 7 | 5 | 7 | 3 | 3 | 2 |
|  | 1.5 | / | / | / | 1 | / | 1 | / | / | / |
| Blood (Ery/μl) | Neg | 14 | 15 | 14 | 10 | 10 | 10 | 5 | 5 | 5 |
|  | ca.70 | 1 | / | 1 | / | / | / | / | / | / |
| Protein (g/L) | Neg | 15 | 15 | 14 | 10 | 10 | 10 | 4/5 | 5/5 | 4/5 |
|  | Trace | / | / | 1 |  |  |  | 1/5 | / | 1/5 |
| Nitrite | Neg | 15 | 15 | 12 | 10 | 10 | 10 | 5 | 5/5 | 5/5 |
| Leukocytes (Leuko/μL) | Neg | 10 | 9 | 8 | 8 | 8 | 8 | 2 | 1 | 3 |
|  | ca.15 | 4 | 6 | 5 | 2 | 2 | 2 | 1 | 4 | 1 |
|  | ca.70 | 1 | / | 2 | / | / | / | 1 | / | 1 |
|  | ca.125 | / | / | / | / | / | / | 1 | / | / |
| Glucose (mmol/L) | Neg | 15 | 15 | 15 | 10 | 10 | 10 | 5 | 5 | 5 |
| Microalbumin (g/L) | Neg | 1 | 2 | / | / | 2 | / | / | / | 1 |
|  | 0.15 | 14 | 13 | 14 | 10 | 8 | 10 | 4 | 5 | 3 |
|  | >0.15 | / | / | 1 | / | / | / | 1 | / | 1 |
| Specific gravity | <=1.005 | 8 | 9 | 7 | 10 | 8 | 10 | 2 | 4 | 5 |
|  | 1.010 | 7 | 6 | 7 | / | 1 | / | 2 | 1 | / |
|  | 1.015 | / | / | 1 | / | 1 | / | 1 | / | / |
| pH | 6.0 | / | / | / | / | / | 1 | / | / | / |
|  | 6.5 | / | / | / | 1 | 1 | / | / | / | 1 |
|  | 7.0 | / | / | / | 4 | 6 | 3 | / | / | 1 |
|  | 7.5 | 6 | 8 | 4 | 5 | 3 | 4 | 4 | 2 | 3 |
|  | 8.0 | 8 | 7 | 9 | / | / | 2 | 1 | 3 | /^▲^ |
|  | 8.5 | 1 | / | 2 | / | / | / | / | / |  |
| Vitamin C (mmol/L) | 0.0 | 15 | 15 | 15 | 10 | 9 | 10 | 5 | 5 | 5 |
|  | 1.4 | / | / | / | / | 1 | / | / | / | / |

Note: “Normal” and “Neg” indicate no abnormality in the indicator. ^“▲”^indicates a statistically significant difference at p <0.05 when compared to the Flu vaccine group.

**Supplementary table 10 The urinalysis of female SD rats treated with Flu-CoV2 vaccine in Study 2.**

| **Parameter** | | **D0** | | | **D74** | | | **D85** | | |
| --- | --- | --- | --- | --- | --- | --- | --- | --- | --- | --- |
|  |  | **Control** | **Flu vaccine+Saline** | **Flu+** **Flu-CoV2 vaccine** | **Control** | **Flu vaccine** | **Flu+** **Flu-CoV2 vaccine** | **Control** | **Flu vaccine+Saline** | **Flu+** **Flu-CoV2 vaccine** |
| Number of animals | | 15 | 15 | 15 | 10 | 10 | 10 | 5 | 5 | 5 |
| Urobilinogen (μmol/L) | Normal | 15 | 15 | 15 | 10 | 10 | 10 | 5 | 5 | 5 |
| Bilirubin (μmol/L) | Neg | 15 | 15 | 15 | 10 | 10 | 10 | 5 | 5 | 5 |
| Ketone bodies (mmol/L) | Neg | 15 | 15 | 15 | 10 | 10 | 10 | 5 | 5 | 5 |
| Blood (Ery/μl) | Neg | 15 | 15 | 15 | 10 | 10 | 9 | 5 | 5 | 5 |
|  | ca.10 | / | / | / | / | / | 1 | / | / | / |
| Protein (g/L) | Neg | 15 | 15 | 15 | 10 | 10 | 10 | 5 | 5 | 5 |
| Nitrite | Neg | 14 | 15 | 14 | 10 | 10 | 10 | 5 | 5 | 5 |
|  | Pos | 1 | / | 1 | / | / | / | / | / | / |
| Leukocytes (Leuko/μL) | Neg | 15 | 15 | 15 | 10 | 10 | 10 | 4 | 5 | 5 |
|  | ca.15 | / | / | / | / | / | / | 1 | / | / |
| Glucose (mmol/L) | Neg | 15 | 15 | 15 | 10 | 10 | 10 | 5 | 5 | 5 |
| Microalbumin (g/L) | Neg | 11 | 11 | 10 | 8 | 9 | 7 | / | 3 | 2 |
|  | 0.15 | 4 | 4 | 5 | 2 | 1 | 3 | 5 | 2 | 3 |
| Specific gravity | <=1.005 | 15 | 13 | 13 | 10 | 9 | 10 | 5 | 4 | 3 |
|  | 1.010 | / | 1 | 2 | / | 1 | / | / | 1 | 2 |
|  | 1.015 | / | 1 | / | / | / | / | / | / | / |
| pH | 5.5 | / | / | / | / | / | 1 | / |  |  |
|  | 6.0 | / | / | / | / | 1 | 2 | / | 1 | / |
|  | 6.5 | 1 | 2 | / | 2 | 6 | 1 | 2 | / | 3 |
|  | 7.0 | / | 2 | 1 | 3 | 2 | 2 | 1 | 2 | / |
|  | 7.5 | 10 | 7 | 9 | 5 | 1 | 4 | 2 | 2 | 2 |
|  | 8.0 | 4 | 4 | 5 | / | / | / | / | / | / |
| Vitamin C (mmol/L) | 0.0 | 15 | 15 | 15 | 10 | 9 | 10 | 5 | 4 | 2 |
|  | 0.6 | / | / | / | / | 1 | / | / | 1 | 3 |

Note: “Normal” and “Neg” indicate no abnormality in the indicator. “POS” indicates a nitrite test result of any degree of uniform pink.

**Supplementary table 11 The urinalysis of male SD rats treated with Flu-CoV2 vaccine in Study 3.**

| **Parameter** | | **D0** | | | | **D101** | | | | **D113** | | | |
| --- | --- | --- | --- | --- | --- | --- | --- | --- | --- | --- | --- | --- | --- |
|  |  | **Control** | **CoV2 vaccine+Saline** | **CoV2+Flu vaccine** | **CoV2+** **Flu-CoV2 vaccine** | **Control** | **CoV2 vaccine+Saline** | **CoV2+Flu vaccine** | **CoV2+** **Flu-CoV2 vaccine** | **Control** | **CoV2 vaccine+Saline** | **CoV2+Flu vaccine** | **CoV2+** **Flu-CoV2 vaccine** |
| Number of animals | | 15 | 15 | 15 | 15 | 10 | 10 | 10 | 10 | 5 | 5 | 5 | 5 |
| Urobilinogen (μmol/L) | Normal | 15 | 15 | 15 | 15 | 10 | 10 | 10 | 10 | 5 | 5 | 5 | 5 |
| Bilirubin (μmol/L) | Neg | 15 | 15 | 15 | 15 | 10 | 10 | 10 | 10 | 5 | 5 | 5 | 5 |
| Ketone bodies (mmol/L) | Neg | 11 | 9 | 10 | 13 | 2 | 2 | 5 | 2 | 1 | 4 | 4 | 1 |
|  | 0.5 | 4 | 6 | 5 | 2 | 7 | 7 | 5 | 8 | 4 | 1 | 1 | 4 |
|  | 1.5 | / | / | / | / | 1 | 1 | / | / | / | / | / | / |
| Blood (Ery/μl) | Neg | 14 | 14 | 13 | 13 | 10 | 10 | 10 | 10 | 5 | 4 | 5 | 5 |
|  | ca.10 | 1 | 1 | / | 1 | / | / | / | / | / | 1 | / | / |
|  | ca.25 | / | / | 2 | 1 | / | / | / | / | / | / | / | / |
| Protein (g/L) | Neg | 14 | 14 | 13 | 15 | 10 | 10 | 10 | 9 | 2 | 3 | 4 | 4 |
|  | Trace | 1 | 1 | 2 | / | / | / | / | 1 | 3 | 2 | 1 | 1 |
| Nitrite | Neg | 15 | 14 | 14 | 14 | 10 | 10 | 10 | 10 | 5 | 5 | 5 | 5 |
|  | Pos | / | 1 | 1 | 1 | / | / | / | / | / | / | / | / |
| Leukocytes (Leuko/μL) | Neg | 9 | 7 | 9 | 8 | 8 | 8 | 8 | 8 | / | 1 | 3 | 2 |
|  | ca.15 | 5 | 7 | 5 | 6 | 1 | 2 | 2 | 2 | 2 | 4 | 2 | 2 |
|  | ca.70 | 1 | 1 | 1 | 1 | 1 | / | / | / | 2 | / | / | 1 |
|  | ca.125 | / | / | / | / | / | / | / | / | 1 | / | / | / |
| Glucose (mmol/L) | Neg | 15 | 15 | 15 | 15 | 10 | 10 | 10 | 10 | 5 | 5 | 5 | 5 |
| Microalbumin (g/L) | Neg | / | / | / | 1 | / | / | / | 4 |  |  |  |  |
|  | 0.15 | 14 | 14 | 13 | 14 | 10 | 10 | 10 | 5 | 2 | 3 | 4 | 4 |
|  | >0.15 | 1 | 1 | 2 | / | / | / | / | 1 | 3 | 2 | 1 | 1 |
| Specific gravity | <=1.005 | 7 | 8 | 10 | 10 | 10 | 8 | 10 | 10 | 3 | 5 | 4 | 4 |
|  | 1.010 | 8 | 7 | 4 | 5 |  |  |  |  | 2 | / | 1 | 1 |
|  | 1.015 | / | / | 1 | / | / | 2 | / | / | / | / | / | / |
| pH | 6.0 | / | / | / | / | 1 | / | 1 | / | / | / | / | / |
|  | 6.5 | / | / | / | / | 2 | / | 1 | 1 | / | / | / | / |
|  | 7.0 | 1 | / | / | 2 | 1 | 6 | 1 | 2 | / | 2 | / | / |
|  | 7.5 | 3 | 5 | 5 | 5 | 6 | 4 | 5 | 6 | 4 | 1 | 2 | 3 |
|  | 8.0 | 8 | 8 | 7 | 8 | / | / | 2 | 1 | 1 | 2 | 3 | 2 |
|  | 8.5 | 3 | 2 | 3 | / | / | / | / | / | / | / | / | / |
| Vitamin C (mmol/L) | 0.0 | 13 | 15 | 15 | 14 | 10 | 9 | 10 | 10 | 5 | 5 | 5 | 5 |
|  | 0.6 | 2 | / | / | 1 | / | / | / | / | / | / | / | / |
|  | 1.4 | / | / | / | / | / | 1 | / | / | / | / | / | / |

Note: “Normal” and “Neg” indicate no abnormality in the indicator. “POS” indicates a nitrite test result of any degree of uniform pink.

**Supplementary table 12 The urinalysis of female SD rats treated with Flu-CoV2 vaccine in Study 3.**

| **Parameter** | | **D0** | | | | **D101** | | | | **D113** | | | |
| --- | --- | --- | --- | --- | --- | --- | --- | --- | --- | --- | --- | --- | --- |
|  |  | **Control** | **CoV2 vaccine+Saline** | **CoV2+Flu vaccine** | **CoV2+** **Flu-CoV2 vaccine** | **Control** | **CoV2 vaccine+Saline** | **CoV2+Flu vaccine** | **CoV2+** **Flu-CoV2 vaccine** | **Control** | **CoV2 vaccine+Saline** | **CoV2+Flu vaccine** | **CoV2+** **Flu-CoV2 vaccine** |
| Number of animals | | 15 | 15 | 15 | 15 | 10 | 10 | 10 | 10 | 5 | 5 | 5 | 5 |
| Urobilinogen (μmol/L) | Normal | 15 | 15 | 15 | 15 | 10 | 10 | 10 | 10 | 5 | 5 | 5 | 5 |
| Bilirubin (μmol/L) | Neg | 15 | 15 | 15 | 15 | 10 | 10 | 10 | 10 | 5 | 5 | 5 | 5 |
| Ketone bodies (mmol/L) | Neg | 15 | 15 | 14 | 15 | 10 | 10 | 9 | 10 | 5 | 5 | 5 | 5 |
|  | 0.5 | / | / | 1 | / | / | / | 1 | / | / | / | / | / |
| Blood (Ery/μl) | Neg | 14 | 13 | 15 | 14 | 10 | 10 | 9 | 10 | 5 | 5 | 5 | 4 |
|  | ca.10 | / | 1 | / | / | / | / | / | / | / | / | / | / |
|  | ca.25 |  | / | / | / | / | / | 1 | / | / | / | / | 1 |
|  | ca.80 | / | 1 | / | 1 | / | / | / | / | / | / | / | / |
|  | ca.200 | 1 | / | / | / | / | / | / | / | / | / | / | / |
| Protein (g/L) | Neg | 14 | 15 | 14 | 15 | 10 | 10 | 10 | 10 | 5 | 5 | 5 | 4 |
|  | Trace | 1 | / | 1 | / | / | / | / | / | / | / | / | 1 |
| Nitrite | Neg | 12 | 14 | 15 | 14 | 10 | 10 | 10 | 10 | 5 | 5 | 5 | 5 |
|  | Pos | 3 | 1 | / | 1 | / | / | / | / | / | / | / | / |
| Leukocytes (Leuko/μL) | Neg | 15 | 15 | 14 | 15 | 10 | 10 | 10 | 10 | 4 | 5 | 4 | 4 |
|  | 15 | / | / | 1 | / | / | / | / | / | 1 | / | 1 | / |
|  | 70 | / | / | / | / | / | / | / | / | / | / | / | 1 |
| Glucose (mmol/L) | Neg | 15 | 15 | 15 | 15 | 10 | 10 | 10 | 10 | 5 | 5 | 5 | 5 |
| Microalbumin (g/L) | Neg | 6 | 11 | 9 | 8 | 10 | 8 | 6 | 9 | 1 | 3 | 3 | 3 |
|  | 0.15 | 8 | 4 | 5 | 7 | / | 2 | 4 | 1 | 4 | 2 | 2 | 1 |
|  | >0.15 | 1 | / | 1 | / | / | / | / | / | / | / | / | 1 |
| Specific gravity | <=1.005 | 12 | 13 | 14 | 14 | 10 | 9 | 10 | 10 | 4 | 4 | 5/5 | 4 |
|  | 1.010 | 3 | 2 | 1 | 1 | / | 1 | / | / | 1 | 1 | / | 1 |
| pH | 5.5 | / | / | / | / | / | / | / | 1 | / | / | / | / |
|  | 6.0 | / | / | 1/ | / | 1 | 1 | 3 | 1 | 1 | 1 | 1 | 2 |
|  | 6.5 | 2 | 2 | / | 1 | 1 | 6 | 3 | 2 | 1 | 3 | 1 | 1 |
|  | 7.0 | / | 1 | 1 | / | 3 | 2 | / | 3 | / | 1 | / | / |
|  | 7.5 | 5 | 5 | 6 | 4 | 5 | 1 | 4 | 3 | 2 | / | 3 | 2 |
|  | 8.0 | 7 | 4 | 6 | 10 | / | / | / | / | 1 | / | / | / |
|  | 8.5 | 1 | 3 | 1 | / | / | / | / | / | / | / | / | / |
| Vitamin C (mmol/L) | 0.0 | 14 | 15 | 15 | 15 | 10 | 9 | 10 | 10 | 5 | 4 | 5 | 5 |
|  | 0.6 | 1 | / | / | / | / | 1 | / | / | / | 1 | / | / |

Note: “Normal” and “Neg” indicate no abnormality in the indicator. “POS” indicates a nitrite test result of any degree of uniform pink.
